# Association of Genome-Wide Polygenic Scores for Multiple Psychiatric and Common Traits in Preadolescent Youths at Risk of Suicide

**DOI:** 10.1001/jamanetworkopen.2021.48585

**Published:** 2022-02-21

**Authors:** Yoonjung Yoonie Joo, Seo-Yoon Moon, Hee-Hwan Wang, Hyeonjin Kim, Eun-Ji Lee, Jong Hun Kim, Jonathan Posner, Woo-Young Ahn, Incheol Choi, Jae-Won Kim, Jiook Cha

**Affiliations:** 1 Department of Psychology, Seoul National University, Seoul, Republic of Korea; 2 Institute of Data Science, Korea University, Seoul, Republic of Korea; 3 College of Liberal Studies, Seoul National University, Seoul, Republic of Korea; 4 Department of Brain and Cognitive Sciences, Seoul National University, Seoul, Republic of Korea; 5 Department of Neurology, Dementia Center, Stroke Center, National Health Insurance Service Ilsan Hospital, Goyang, Republic of Korea; 6 Department of Psychiatry, Columbia University Medical Center, New York, New York; 7 Department of Child and Adolescent Psychiatry, Seoul National University Hospital, Seoul, Republic of Korea; 8 Artificial Intelligence Institute, Seoul National University, Seoul, Republic of Korea

## Abstract

**Importance:**

Suicide is the second leading cause of death among youths worldwide, but no available means exist to identify the risk of suicide in this population.

**Objective:**

To assess whether genome-wide polygenic scores for psychiatric and common traits are associated with the risk of suicide among preadolescent children and to investigate whether and to what extent the interaction between early life stress (a major environmental risk factor) and polygenic factors is associated with suicidal thoughts and behaviors among youths.

**Design, Setting, and Participants:**

This cohort study analyzed the genotype-phenotype data of 11 869 preadolescent children aged 9 to 10 years from the Adolescent Brain and Cognitive Development study. Data were collected from September 1, 2016, to October 21, 2018, and analyzed from August 1, 2020, to January 3, 2021. Using machine learning approaches, genome-wide polygenic scores of 24 complex traits were estimated to investigate their phenome-wide associations and utility for assessing risk of suicidal thoughts and behaviors (suicidal ideation [active, passive, and overall] and suicide attempt).

**Main Outcomes and Measures:**

Genome-wide polygenic scores were used to measure 24 traits, including psychiatric disorders, cognitive capacity, and personality and psychological characteristics. The Child Behavior Checklist was used to measure early life stress, and the Family Environment Scale was used to assess family environment. Suicidal ideation and suicide attempts were derived from the computerized version of the Kiddie Schedule for Affective Disorders and Schizophrenia.

**Results:**

Among 11 869 preadolescent children in the US, complete data for phenotypic outcomes, genotypes, and covariates were available for 7140 participants in the multiethnic cohort (mean [SD] age, 9.9 [0.6] years; 3588 girls [50.3%]), including 925 participants with suicidal ideation and 63 participants with suicide attempts. Among those 7140 participants, 729 had African ancestry (self-reported race or ethnicity: 569 Black, 71 Hispanic, and 89 other), 276 had admixed American ancestry (self-reported race or ethnicity: 265 Hispanic, 3 White, and 8 other), 150 had East Asian ancestry (self-reported race or ethnicity: 67 Asian, 18 Hispanic, and 65 other), 5718 had European ancestry (self-reported race or ethnicity: 7 Asian, 39 Black, 1142 Hispanic, 3934 White, and 596 other), and 267 had other ancestries (self-reported race or ethnicity: 70 Asian, 13 Black, 126 Hispanic, 48 White, and 10 other). Three genome-wide polygenic scores were significantly associated (false discovery rate *P* < .05) with suicidal thoughts and behaviors among all participants: attention-deficit/hyperactivity disorder (odds ratio [OR], 1.12; 95% CI, 1.05-1.21; *P* = .001), schizophrenia (OR, 1.50; 95% CI, 1.17-1.93; *P* = .002), and general happiness (OR, 0.89; 95% CI, 0.83-0.96; *P* = .002). In the analysis including only children with European ancestry, 3 additional genome-wide polygenic scores with false discovery rate significance were associated with suicidal thoughts and behaviors: autism spectrum disorder (OR, 1.18; 95% CI, 1.06-1.31; *P* = .002), major depressive disorder (OR, 1.12; 95% CI, 1.04-1.21; *P* = .003), and posttraumatic stress disorder (OR, 1.12; 95% CI, 1.04-1.21; *P* = .004). A significant interaction between genome-wide polygenic scores and environment was found, with genetic risk factors for autism spectrum disorder and the level of early life stress associated with increases in the risk of overall suicidal ideation and overall suicidal thoughts and behaviors (OR, 1.20; 95% CI, 1.07-1.35; *P* = .002). A machine learning model using multitrait genome-wide polygenic scores and additional self-reported questionnaire data (Child Behavior Checklist and Family Environment Scale) produced a moderately accurate estimate of overall suicidal thoughts and behaviors (area under the receiver operating characteristic curve [AUROC], 0.77; 95% CI, 0.73-0.81; accuracy, 0.67) and suicidal ideation (AUROC, 0.76; 95% CI, 0.72-0.80; accuracy, 0.66) among children with European ancestry only. Among all children in the multiethnic cohort, the integrated model also outperformed the baseline model in estimating the risk of overall suicidal thoughts and behaviors (AUROC, 0.71; 95% CI, 0.67-0.75; accuracy, 0.68) and suicidal ideation (AUROC, 0.75; 95% CI, 0.71-0.78; accuracy, 0.67).

**Conclusions and Relevance:**

In this cohort study of preadolescent youths in the US, higher genome-wide polygenic scores for psychiatric disorders, such as attention-deficit/hyperactivity disorder, autism spectrum disorder, posttraumatic stress disorder, and schizophrenia, were significantly associated with a greater risk of suicidal ideation and suicide attempt. The findings and quantitative models from this study may help to identify children with a high risk of suicide, potentially assisting with early screening, intervention, and prevention.

## Key Points

**Question:**

Are genome-wide polygenic scores for specific psychiatric and common traits associated with high risk of suicide among preadolescent youths?

**Findings:**

In this cohort study of 11 869 preadolescent youths in the US, multiple genome-wide polygenic scores were significantly associated with suicidal thoughts and behaviors (ideation or attempts); specific genome-wide polygenic scores associated with the risk of suicide included attention-deficit/hyperactivity disorder, general happiness, and posttraumatic stress disorder.

**Meaning:**

These results suggest that the genomic approach may be useful for identifying children at high risk for suicidal thoughts and behaviors.

## Teaser

This cohort study assesses the association between genome-wide polygenic scores for psychiatric and common traits and the risk of suicide among preadolescent children in the US.

## Introduction

Every minute, 2.6 persons in the US attempt suicide, and one of them succeeds every 11 minutes.^1^ Among youths, suicide is the second leading cause of death worldwide; the suicide rate has not been reduced in decades.^1,2^ Identifying a child at risk of suicide is challenging, and existing approaches^3,4^ have low predictive validity and limited practical utility.^5^ Existing prediction algorithms either do not use genetic or environmental factors to efficiently identify individuals at high risk for suicide or mainly focus on higher-risk subpopulations, such as military service members, veterans, and health care systems serving adult civilians, limiting the algorithms’ generalizability among pediatric cohorts or health care systems serving children.

Twin and family studies^6,7^ have found that suicidal behaviors, including suicidal ideation and suicide attempt, are significantly associated with genetic factors, with estimated heritability ranging from 30% to 55%.^6,8^ A previous study^9^ suggested that genetic factors associated with suicide risk, perhaps having implications for diatheses for suicidal ideation and suicide attempt, may interact with environmental factors, such as stressful events. However, to our knowledge, no studies examining the genetic factors associated with suicidal thoughts and behaviors among youths or the interactions between genetic and environmental factors have been published.

According to studies of adults, the genetic architecture of suicidal behaviors is polygenic^10,11^ and associated with the cumulative effects of numerous single-nucleotide variants (SNVs), each with minuscule associations. Previous genome-wide association studies (GWASs) have revealed several loci associated with completed suicide,^12,13^ suicide attempt,^14,15^ and suicidal ideation.^16,17,18,19^ However, none of the loci has been replicated across studies. Based on GWAS findings, genome-wide polygenic scores can be used to integrate the cumulative effects of genome-wide SNVs^20,21^ and have emerged as a potential tool for efficiently predicting the likelihood of a complex trait from a genomic perspective.^22,23^ The genome-wide polygenic scores can be used to stratify individuals at higher biological risk or to provide insights into the possible genetic overlap among complex traits, potentially enabling personalized medicine.^24,25,26^

Genetic predisposition to suicidal behaviors has been reported to interact with environmental factors^11^ such as early life stress,^27,28^ potentially via epigenetic mechanisms.^29,30^ Investigating whether and to what extent early life stress and genetic factors act synergistically on suicidal thoughts and behaviors among youths will offer needed insight into the biological factors associated with suicide and may provide actionable targets for intervention. However, to our knowledge, no studies have investigated the interaction between genetic and environmental factors or the utility of genetic factors for predicting suicidal thoughts and behaviors among youths. In this cohort study, we investigated whether and to what extent genome-wide polygenic scores^24,25^ for common traits and psychiatric disorders interacted with early life stress and were associated with the risk of suicide among preadolescent children in the US.

## Methods

### Study Design and Participants

**Figure 1.**
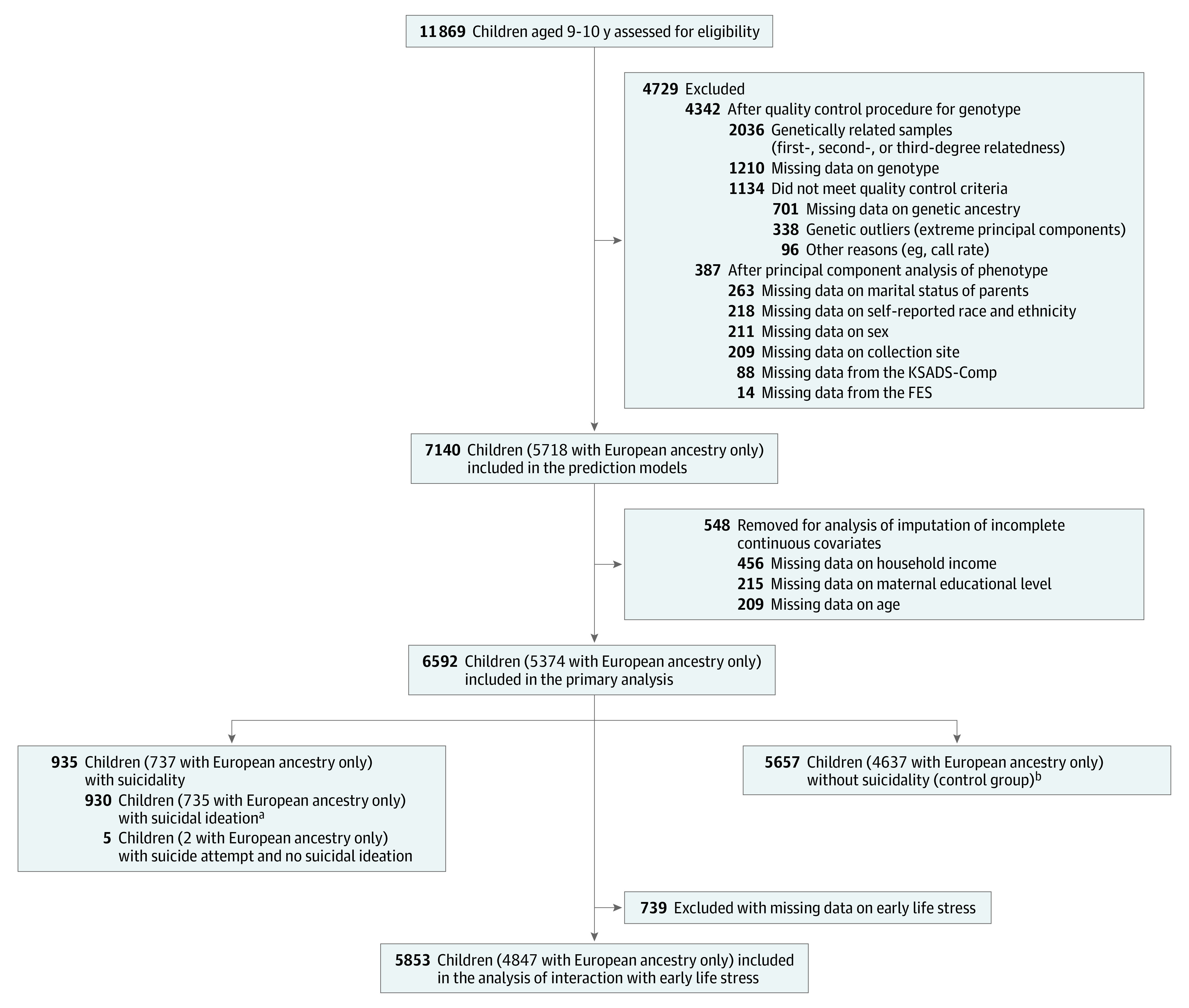
Study Flow Diagram The study initially assessed 11 869 preadolescent children aged 9 to 10 years recruited from the Adolescent Brain and Cognitive Development study. After an initial quality control assessment, complete data including phenotypic outcome, genotype, and covariate data were available for 7140 children in the multiethnic cohort (5718 of whom had European ancestry only). Continuous variables for those individuals were imputed and used in the models. For the primary association analysis, individuals with missing continuous data were removed, and the integrative multimodal data of 6592 children in the multiethnic cohort (5374 of whom had European ancestry only) were included. FES indicates Family Environment Scale; and KSADS-Comp, Kiddie Schedule for Affective Disorders and Schizophrenia, computerized version. ^a^Includes 59 children in the multiethnic cohort (43 of whom had European ancestry only) who attempted suicide. ^b^Includes 1652 children in the multiethnic cohort (1351 of whom had European ancestry only) who were missing data from the KSADS-Comp.

This cohort study included 11 869 preadolescent children across 21 sites in the US who were recruited from the Adolescent Brain and Cognitive Development (ABCD) cohort between September 1, 2016, and October 21, 2018.^31^ We analyzed data from the ABCD study, data release 2.0 (excluding information about genetic race and ethnicity from the ABCD study, data release 3.0), including data on children with African ancestry (comprising children with self-reported Black, Hispanic, or other race or ethnicity), admixed American ancestry (comprising children with self-reported Hispanic, White, or other race or ethnicity), East Asian ancestry (comprising children with self-reported Asian, Hispanic, or other race or ethnicity), European ancestry (comprising children with self-reported Asian, Black, Hispanic, White, or other race or ethnicity), and other ancestry (comprising children with self-reported Asian, Black, Hispanic, White, or other race or ethnicity). Data were analyzed from August 1, 2020, to January 3, 2021. Participants with missing data on study variables were removed from the sample data set. The sample sizes for each analysis are shown in Figure 1. Full details of the study measures and samples can be found elsewhere.^32^ Ethical approval for the study was obtained from the institutional review board of Seoul National University. Written informed consent was obtained from both parents and children. This study followed the Strengthening the Reporting of Observational Studies in Epidemiology (STROBE) reporting guideline for cohort studies.

### Genotype Data

Saliva samples of participants were collected and genotyped at the Rutgers University Cell and DNA Repository using the Affymetrix SmokeScreen array (Thermo Fisher Scientific) consisting of 733 293 SNVs. After removing SNVs with a genotype call rate (ie, the proportion of genotypes per marker with nonmissing data) less than 95%, a sample call rate (ie, the proportion of called SNVs in the sample divided by the total number of SNVs in the data set) less than 95%, and minor allele frequency lower than 1%, raw genotypes were imputed using the 1000 Genomes phase 3, version 5, reference panel (1000 Genomes Project) from the Michigan Imputation Server^33^ and phased using the Eagle2, version 2.4, algorithm^34^ (1000 Genomes Project; 12 046 090 total variants). An additional quality control process was performed to remove SNVs with an INFO score (ie, imputation quality score of 0-1, with values closer to 1 indicating an SNV has been imputed with higher certainty) less than 0.4, a genotype call rate less than 95%, a Hardy-Weinberg equilibrium *P* < 1 × 10^−20^ for ethnically diverse populations, sample missingness greater than 5%, and minor allele frequency lower than 0.5%. We also removed samples with extreme heterozygosity (ie, samples with an *F* coefficient >3 SDs higher than the population mean).

The study samples had complex genetic structures owing to related family members, diverse genetic ancestries, and admixed samples innate to the US population. We used the PC-AiR method^35^ to estimate ancestrally informative principal components of the genotypes robust to the related pedigree structure, and we used the PC-Relate method^36^ to provide an accurate estimate of recent genetic relatedness measures from the population with admixed ancestry. The details of the quality control procedure are available in eFigure 1 in the Supplement. The results reported in this article were based on genotype data (11 301 999 total variants) from 7426 genetically unrelated samples (after the quality control procedure was performed). The first 10 principal components of final genotype data were used to calculate the genome-wide polygenic scores.

### Construction of Genome-Wide Polygenic Scores

For generation of genome-wide polygenic scores, we selected 24 psychiatric and common traits that were known to be associated with suicidal thoughts and behaviors and had publicly available GWAS summary statistics, including personality, cognitive, and psychological traits as well as psychiatric disorders that were known to be broadly associated with suicidal thoughts and behaviors, including general happiness,^37,38^ insomnia,^39^ depression,^40^ risk behaviors,^41^ risk tolerance,^42^ educational attainment,^43,44^ cognitive performance,^41,43^ snoring,^45^ worry,^46^ IQ,^43^ cannabis use,^47,48^ alcoholic drinks per week,^49^ smoking status,^50^ attention-deficit/hyperactivity disorder (ADHD),^51^ autism spectrum disorder (ASD),^52^ major depressive disorder (MDD),^53^ schizophrenia,^54^ bipolar disorder,^55^ posttraumatic stress disorder (PTSD),^56^ and Alzheimer disease.^57^ For the trait of general happiness, 4 genome-wide polygenic scores were built and tested for the study. Different questionnaires were used to discern participants’ level of subjective well-being. Questions included (1) 2 different questions about life satisfaction and/or positive affect, comprising “how happy are you in general?” (trait named *general happiness*) and “how satisfied are you with your life as a whole?” (trait named *subjective well-being*); (2) “how happy are you with your health in general?” (trait named *general happiness with own health*); and (3) “to what extent do you feel your life to be meaningful?” (trait named *belief that own life is meaningful*). All of the GWAS summary statistics of the traits examined in the study are publicly available (eTable 1 in the Supplement).

We performed clumping and pruning of SNVs using PRSice-2 software, version 3.0 (GNU General Public License),^58^ with a clumping window of 500 kb, a clumping *r*^2^ of 0.2 based on the 1000 Genomes cosmopolitan panel (1000 Genomes Project), and no thresholding of *P* value significance for the summary statistics because we wanted to fully incorporate the effects of all SNVs. The genome-wide polygenic score for each individual was then computed as the sum of their SNVs, adjusting for the first 10 genotype principal components, with each SNV weighted by the effect in the discovery samples.^58^ Because most of the summary statistics were derived from the European population, we selected 5749 children with European ancestry only (determined using the fastSTRUCTURE algorithm^59^) from the ABCD study (data release 3.0) for further analysis.

### Outcomes and Measures

Data on suicidal ideation (active, passive, and overall) and suicide attempt were derived from the computerized version of the Kiddie Schedule for Affective Disorders and Schizophrenia (KSADS-Comp).^60,61^ Passive suicidal ideation was defined as wanting to be dead, and active ideation was defined as considering suicide with specific methods or plans (eTable 2 in the Supplement). Children without suicidal ideation or suicide attempts served as the primary control group; children with no active or past records of KSADS-Comp diagnoses (1652 participants in the multiethnic cohort, 1351 of whom had European ancestry only) were included in the secondary healthy control group. Among parent and child reports of KSADS-Comp diagnoses, we used the version that reported more severe symptoms or diagnoses. Variables considered for inclusion in the classification models are described in eMethods in the Supplement.

### Machine Learning Modeling

For assessing suicide risk using genome-wide polygenic scores, we trained multivariate logistic regression, random forest, and elastic net models using the caret package in R software, version 3.6.3 (R Foundation for Statistical Computing). The following genetic data and self-reported questionnaires were used to build the model: 24 multitrait genome-wide polygenic scores, sociodemographic information (sex, marital status of parents, family income, maternal educational attainment, study site, and self-reported ethnicity for additional multiethnic analysis), psychological observations (measured by the Child Behavior Checklist [CBCL]), family environment factors (measured by the Family Environment Scale), and early life stress scores. We performed median imputation on incomplete continuous covariates, such as family income and maternal educational attainment, from 548 children. We hypothesized that multitrait genome-wide polygenic scores would account for the multidimensional genetic predisposition to suicidal behaviors. For model training and evaluation, data were split into training (80%) and test (20%) sets, in which controls were randomly undersampled. Within the training set, 5-fold stratified cross-validation was performed with grid search to find the optimal hyperparameters. Model performance was evaluated using the held-out replication set by calculating the area under the receiver operating characteristic curve (AUROC), 95% CI, positive predictive value, negative predictive value, and accuracy.

### Statistical Analysis

After completion of the quality control procedure and construction of genome-wide polygenic scores, complete data for phenotypic outcomes, genome-wide polygenic scores, and covariate data were available for 6592 children in the multiethnic cohort and used for statistical analysis (Figure 1). We examined the associations between 24 genome-wide polygenic scores and suicidal phenotypes among all 6592 children in the multiethnic cohort and 5374 of those children who had European ancestry only. Both analyses used logistic regression models adjusted for sex, sample collection site, family income, maternal educational attainment, and self-reported race and ethnicity. We used a false discovery rate to control for multiple testing. Data were analyzed using R software, version 3.6.1. The threshold for statistical significance was false discovery rate *P* < .05.

## Results

This cohort study included 11 869 participants from the ABCD study, a nationwide prospective cohort of preadolescent children aged 9 to 10 years across 21 sites in the US who were recruited between September 1, 2016, and October 21, 2018. After performing the quality control procedure, complete data from 7140 children in the multiethnic cohort (mean [SD] age, 9.9 [0.6] years; 3588 girls [50.3%] and 3552 boys [49.7%]) were available for inclusion in our models. Among those 7140 participants, 729 had African ancestry (self-reported race or ethnicity: 569 Black, 71 Hispanic, and 89 other), 276 had admixed American ancestry (self-reported race or ethnicity: 265 Hispanic, 3 White, and 8 other), 150 had East Asian ancestry (self-reported race or ethnicity: 67 Asian, 18 Hispanic, and 65 other), 5718 had European ancestry (self-reported race or ethnicity: 7 Asian, 39 Black, 1142 Hispanic, 3934 White, and 596 other), and 267 had other ancestries (self-reported race or ethnicity: 70 Asian, 13 Black, 126 Hispanic, 48 White, and 10 other). After excluding continuous variables with missing data, 6592 children in the multiethnic cohort (5374 of whom had European ancestry only; mean [SD] age, 9.9 [0.6] years; 3480 girls [52.8%] and 3112 boys [47.2%]) were included in the primary analysis, including 935 children with suicidal thoughts or behaviors (930 with suicidal ideation and 64 with suicide attempts identified through KSADS-Comp records) and 5657 children without suicidal thoughts or behaviors (control group) (Figure 1). The case vs control groups included in the primary analysis were significantly different in terms of sex ratio (565 girls among 935 participants [60.4%] vs 2915 girls among 5657 participants (51.5%]; *P* < .001), mean (SD) family income ($94 970 [$76 530] vs $99 232 [$74 559]; *P* < .001), and marital status of parents (595 participants [63.6%] vs 4108 participants [72.6] with married parents; *P* < .001), which were all included as covariates in the analysis (Table 1). We did not find significant demographic differences between all participants in the multiethnic cohort and the subgroup of participants with European ancestry only or between the preimputed and postimputed data used for the models (eTable 3 in the Supplement).

**Table 1.** Sociodemographic Characteristics of Participants From the Adolescent Brain and Cognitive Development Study

Characteristic	No. (%)^a^		Statistic	*P* value
	Children with suicidal thoughts and behaviors (n = 935)	Children without suicidal thoughts and behaviors (n = 5657)		
Sex				
Female	565 (60.4)	2915 (51.5)	26.7^b^	<.001
Male	370 (39.6)	2742 (48.5)	26.7^b^	
Age, mean (SD), mo	118.8 (7.4)	119.0 (7.3)	−0.6^c^	.53
Annual household income bracket, mean (SD)^d^	7.13 (2.3)	7.46 (2.3)	4.0^c^	<.001
Parents currently married	595 (63.6)	4108 (72.6)	40.5^b^	<.001
Maternal educational attainment, mean (SD), y	16.87 (2.4)	16.95 (2.6)	0.9^c^	.38
Race and ethnicity^e^				
Asian	23 (2.5)	102 (1.8)	1.52^c^	.22
Black	84 (9.0)	445 (7.9)	1.21^c^	.27
Hispanic	189 (20.2)	1229 (21.7)	1.00^c^	.32
White	515 (55.1)	3294 (58.2)	3.13^c^	.08
Other	124 (13.3)	587 (10.4)	6.65^c^	.01
Participants at collection site with largest sample	93 (9.9)	543 (9.6)	30.4^b^	.09

^a^ Includes 6592 preadolescent children in the US with complete data on phenotypic outcomes, genotypes, and covariates.

^b^ χ^2^ statistic.

^c^ *t* statistic.

^d^ Income brackets ranged from 1 to 10, with 1 indicating less than $5000, 2 indicating $5000 to $11 999, 3 indicating $12 000 to $15 999, 4 indicating $16 000 to $24 999, 5 indicating $25 000 to $34 999, 6 indicating $35 000 to $49 999, 7 indicating $50 000 to $74 999, 8 indicating $75 000 to $99 999, 9 indicating $100 000 to $199 999, and 10 indicating $200 000 or greater.

^e^ Self-reported race and ethnicity rather than genetic ancestry is included because this information better described participant demographic characteristics, which were initially obtained at data collection. Genetic ancestry data were acquired after the additional analysis of ancestry determination was performed during the quality control process.

Our primary analysis examined the association between 24 genome-wide polygenic scores and suicidal thoughts and behaviors among the 935 children with phenotypes for suicidal thoughts and behaviors and 5657 children in the control group (Table 2; eTable 4 in the Supplement). The genome-wide polygenic scores for ADHD had the most significant association with phenotypes for suicidal thoughts and behaviors, revealing that a higher genome-wide polygenic score for ADHD was significantly associated with a greater likelihood of having all suicidal phenotypes, including overall suicidal ideation (odds ratio [OR], 1.12; 95% CI, 1.05-1.21; *P* = .001), active suicidal ideation (OR, 1.17; 95% CI, 1.06-1.29; *P* = .001), and overall suicidal behaviors (OR, 1.12; 95% 1.04-1.20; *P* = .002) (eFigure 2 in the Supplement). The explained variance (as measured by McFadden pseudo-*R*^2^) of suicide attempts associated with the genome-wide polygenic score for ADHD was approximately 10.0% among all children in the multiethnic cohort and 10.5% among children with European ancestry only. We also observed a significant association between the genome-wide polygenic score for schizophrenia and suicide attempt (OR, 1.50; 95% CI, 1.17-1.93; *P* = .002) among children in the multiethnic cohort; correction for false discovery rate (using the McFadden pseudo-*R*^2^) revealed that this association explained up to 11.8% of the variation in suicide attempt. In addition, we found a significant negative association between the genome-wide polygenic score for general happiness and overall suicidal ideation (OR, 0.89; 95% CI, 0.83-0.96; *P* = .002).

**Table 2.** Association of Genome-Wide Polygenic Scores for 24 Psychiatric and Common Traits With Suicidal Thoughts and Behaviors Among Youths^a^

Ancestry	Outcome	Psychiatric or common trait	OR (95% CI)	*P* value	Pseudo-*R*^2^	Cases, No.
Multiethnic cohort^b^	Overall suicidal ideation (active and passive)	ADHD	1.12 (1.05-1.21)	.001	.023	930
		General happiness	0.89 (0.83-0.96)	.002	.023	930
	Active suicidal ideation	ADHD	1.17 (1.06-1.29)	.001	.037	489
	Suicide attempt	Schizophrenia	1.50 (1.17-1.93)	.002	.118	64
	Overall suicidal thoughts and behaviors (ideation and attempt)	ADHD	1.12 (1.04-1.20)	.002	.023	935
European-only cohort	Overall suicidal ideation (active and passive)	ADHD	1.14 (1.06-1.24)	.001	.029	735
		General happiness	0.89 (0.82-0.96)	.003	.028	735
		MDD	1.12 (1.04-1.21)	.003	.028	735
		PTSD	1.12 (1.04-1.21)	.004	.028	735
	Active suicidal ideation	ADHD	1.19 (1.07-1.32)	.001	.045	374
		ASD	1.18 (1.06-1.31)	.002	.045	374
	Passive suicidal ideation	ADHD	1.15 (1.06-1.25)	.001	.027	620
		General happiness	0.89 (0.82-0.96)	.005	.027	620
		MDD	1.12 (1.03-1.22)	.006	.027	620
		PTSD	1.12 (1.03-1.22)	.006	.026	620
	Suicide attempt	Schizophrenia	1.61 (1.21-2.16)	.001	.132	45
	Overall suicidal thoughts and behaviors (ideation and attempt)	ADHD	1.14 (1.05-1.23)	.001	.029	737
		General happiness	0.89 (0.83-0.96)	.003	.029	737
		MDD	1.12 (1.04-1.21)	.003	.029	737
		PTSD	1.12 (1.04-1.21)	.004	.028	737

Abbreviations: ADHD, attention-deficit/hyperactivity disorder; ASD, autism spectrum disorder; MDD, major depressive disorder; OR, odds ratio; PTSD, posttraumatic stress disorder.

^a^ Includes 6592 children in the multiethnic cohort (5374 of whom had European ancestry only). The analysis included associations that had an adjusted false discovery rate *P* value of less than .05 (which indicated that 5% of significant associations were false-positive results). Covariates included in the analysis were sex, site of sample collection, family income, maternal educational level, and self-reported race and ethnicity.

^b^ Includes children with African ancestry (self-reported Black, Hispanic, or other race or ethnicity), admixed American ancestry (self-reported Hispanic, White, or other race or ethnicity), East Asian ancestry (self-reported Asian, Hispanic, or other race or ethnicity), European ancestry (self-reported Asian, Black, Hispanic, White, or other race or ethnicity), and other ancestry (self-reported Asian, Black, Hispanic, White, or other race or ethnicity).

**Figure 2.**
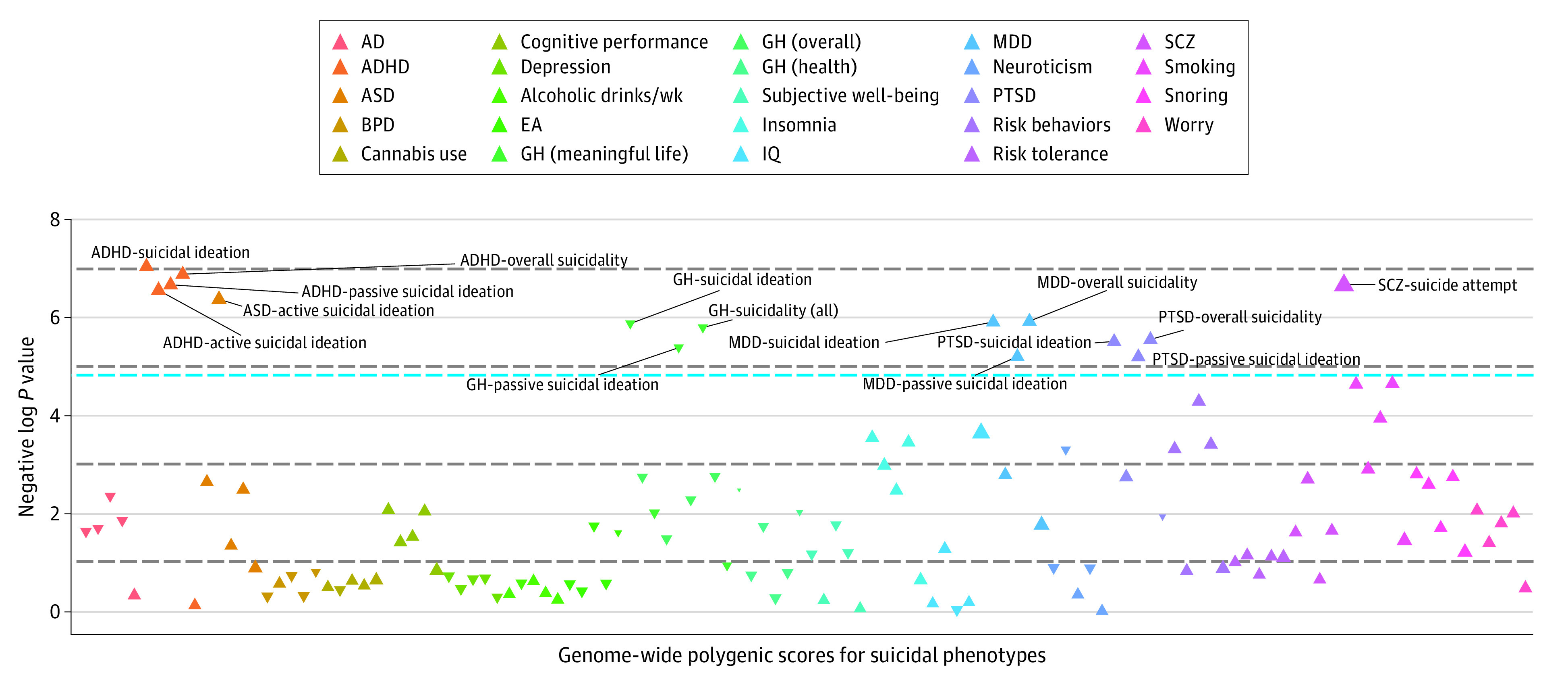
Manhattan Plot of Association Between 24 Genome-Wide Polygenic Scores and Suicidal Thoughts and Behaviors Among Children With European Ancestry Only The analysis included 5374 children aged 9 to 10 years in the multiethnic cohort. The blue line represents a false discovery rate–corrected *P* value of .05. The dotted horizontal lines indicate different levels of statistical significance (negative log *P* values of 1, 3, 5, and 7, from bottom to top of plot). Each triangle represents the effect direction (positive or negative) and the effect size of each association, with inverted triangles indicating negative direction and effect size. The Manhattan plot of the association between 24 genome-wide polygenic scores and suicidal thoughts and behaviors among 6592 children in the multiethnic cohort is available in eFigure 2 in the Supplement. AD indicates Alzheimer disease; ADHD, attention-deficit/hyperactivity disorder; ASD, autism spectrum disorder; BPD, bipolar disorder; EA, educational attainment; GH, general happiness; MDD, major depressive disorder; PTSD, posttraumatic stress disorder; SCZ, schizophrenia.

Results from the analysis of 5374 children with European ancestry only were consistent with the findings from the entire multiethnic cohort. We observed an increased risk of suicide associated with the genome-wide polygenic score for ADHD, with a high risk among several suicidal phenotypes (overall suicidal ideation: OR, 1.14 [95% CI, 1.06-1.24; *P* = .001]; overall suicidal thoughts and behaviors: OR, 1.14 [95% CI, 1.05-1.23; *P* = .001]; passive suicidal ideation: OR, 1.15 [95% CI, 1.06-1.25; *P* = .001]), a positive association between the genome-wide polygenic score for schizophrenia and suicide attempt (OR, 1.61; 95% CI, 1.21-2.16; *P* = .001), and a negative association between the genome-wide polygenic score for general happiness and overall suicidal ideation (OR, 0.89; 95% CI, 0.83-0.96; *P* = .003) (Table 2). Notably, among children with European ancestry only, we found 3 additional psychiatric traits that had significant associations with suicidal phenotypes: ASD (active suicidal ideation: OR, 1.18; 95% CI, 1.06-1.31; *P* = .002), MDD (overall suicidal thoughts and behaviors: OR, 1.12 [95% CI, 1.04-1.21; *P* = .003]; overall suicidal ideation: OR, 1.12 [95% CI, 1.04-1.21; *P* = .003]), and PTSD (overall suicidal thoughts and behaviors: OR, 1.12 [95% CI, 1.04-1.21; *P* = .004]; overall suicidal ideation: OR, 1.12 [95% CI, 1.04-1.21; *P* = .004]) (Figure 2).

Among the 6 traits (ADHD, ASD, general happiness, MDD, PTSD, and schizophrenia) with significant associations between genome-wide polygenic scores and suicidal thoughts and behaviors among participants with European ancestry only, we found that the interaction of early life stress with the genome-wide polygenic score for ASD was significantly associated with active suicidal ideation (OR, 1.20; 95% CI, 1.07-1.35; *P* = .002), overall suicidal ideation (OR, 1.03; 95% CI, 1.03-1.23; *P* = .007), and overall suicidal thoughts and behaviors (OR, 1.03; 95% CI, 1.03-1.23; *P* = .007), suggesting that a higher genome-wide polygenic score for ASD in the presence of early life stress was associated with a greater likelihood of suicidal thoughts and behaviors. Effects were adjusted for the covariates. Bivariate associations between early life stress and suicidal thoughts and behaviors were nonsignificant. When stratified by sex, no significant associations were found.

In the sensitivity analysis, the genome-wide polygenic score for PTSD had the most significant overall association with suicidal phenotypes in the analyses of all participants in the multiethnic cohort (overall suicidal thoughts and behaviors: OR, 1.17; 95% CI, 1.07-1.27; *P* < .001) and participants with European ancestry only (overall suicidal thoughts and behaviors: OR, 1.18; 95% CI, 1.08-1.29; *P* < .001) (eTable 5 in the Supplement). The associations between suicidal thoughts and behaviors and the genome-wide polygenic scores for ADHD, ASD, MDD, and schizophrenia were significant or slightly larger in effect size in the analysis of the healthy control group comprising children who did not have a KSADS-Comp diagnosis for suicidal thought or behaviors. We also found that the genome-wide polygenic score for smoking was significantly associated with overall suicidal ideation (OR, 1.17; 95% CI, 1.06-1.29; *P* = .001).

**Figure 3.**
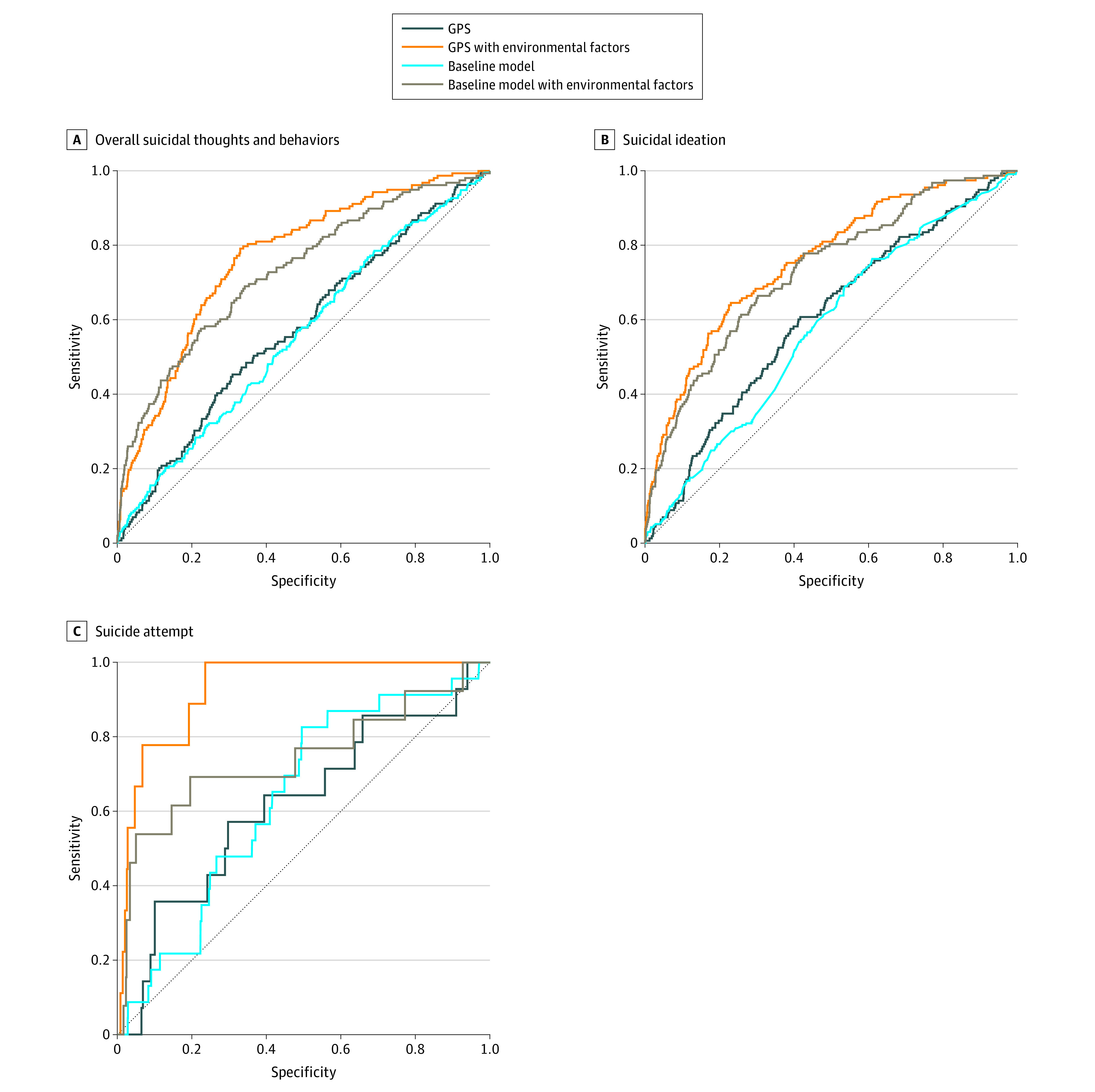
Performance of Machine Learning Models Based on Genome-Wide Polygenic Scores and Cognitive, Psychological, Behavioral, Environmental, and Familial Factors Among Children With European Ancestry Only Receiver operating characteristic (ROC) curves of the models. A total of 5718 children with European ancestry were included in the analysis. A, The area under the ROC (AUROC) of the best model was 0.77 (95% CI, 0.73-0.81; accuracy, 0.67; positive predictive value [PPV], 0.65; negative predictive value [NPV], 0.71). B, The AUROC of the best model was 0.76 (95% CI, 0.72-0.80; accuracy, 0.66; PPV, 0.64; NPV, 0.69). C, The AUROC of the best model was 0.93 (95% CI, 0.87-0.99; accuracy, 0.84; PPV, 0.83; NPV, 0.85) using the elastic net model. The model was also evaluated using data from the entire sample of 7140 children, with the results available in eTable 7 in the Supplement. GPS indicates genome-wide polygenic score.

The multitrait genome-wide polygenic score–based model that included self-reported questionnaire data had the best performance across the suicidal phenotypes. For assessing the risk of overall suicidal thoughts and behaviors among children with European ancestry only, the AUROC of the best model (elastic net) increased to 0.77 (95% CI, 0.73-0.81; accuracy, 0.67) in the balanced held-out test set compared with 0.56 (95% CI, 0.52-0.60; accuracy, 0.56) in the baseline model (Figure 3; eTable 6A in the Supplement). For assessing the risk of suicidal ideation, the model had an AUROC of 0.76 (95% CI, 0.72-0.80; accuracy, 0.66) in the balanced held-out test set. Adding the multitrait genome-wide polygenic scores to the baseline model that included self-reported questionnaire data resulted in AUROC increases of 0.04 for estimating risk of overall suicidal thoughts and behaviors and 0.05 for estimating risk of overall suicidal ideation. The baseline model that included only self-reported questionnaire data (without genome-wide polygenic scores) had moderate performance in assessing risk of overall suicidal thoughts and behaviors (AUROC, 0.73; 95% CI, 0.69-0.78; accuracy, 0.68) and overall suicidal ideation (AUROC, 0.73; 95% CI, 0.69-0.77; accuracy, 0.69). For estimating risk of suicide attempt, the multitrait genome-wide polygenic score–based model that included self-reported questionnaires had an AUROC of 0.93 (95% CI, 0.87-0.99; accuracy, 0.84); however, because the training data set comprised a relatively small sample (90 individuals, including 45 with suicide attempt), the result should be interpreted with caution. Important features in the models included CBCL measures, such as depressive or internalizing symptoms (eTable 7A in the Supplement). For estimating risk of suicide attempt only, the genome-wide polygenic scores for schizophrenia and PTSD had high feature importance based on the CBCL measures. Among all participants in the multiethnic cohort, the results remained unchanged for classifying suicidal thoughts and behaviors (eTable 7B in the Supplement). The integrated model outperformed the baseline model in assessing risk of overall suicidal thoughts and behaviors (AUROC, 0.71; 95% CI, 0.67-0.75; accuracy, 0.68) and suicidal ideation (AUROC, 0.75; 95% CI, 0.71-0.78; accuracy, 0.67).

## Discussion

To our knowledge, this cohort study is the first quantitative assessment of polygenic and environmental factors associated with suicidal thoughts and behaviors among a large nationally representative sample of preadolescent children. We found that suicidal thoughts and behaviors were positively associated with the genome-wide polygenic scores for ADHD, ASD, MDD, PTSD, and schizophrenia and negatively associated with the genome-wide polygenic score for general happiness among all children in the multiethnic cohort and children with European ancestry only. We also found significant genetic and environmental interactions between the genome-wide polygenic score for ASD and early life stress (a known risk factor for suicidal thoughts and behaviors among youths^29^), which together acted cumulatively. In addition, the inclusion of multiple genome-wide polygenic scores combined with data from self-reported questionnaires (ie, the CBCL and the Family Environment Scale) provided moderately accurate identification of preadolescent youths with suicidal thoughts and behaviors.

We identified significant associations between suicidal thoughts and behaviors among youths and the genetic components of 1 common trait (general happiness) and 5 psychiatric disorders (ADHD, ASD, MDD, PTSD, and schizophrenia) that were particularly relevant to childhood psychopathological characteristics. For the 5 significantly associated psychiatric traits, a higher genome-wide polygenic score was associated with a greater likelihood of suicidal behaviors; for general happiness, a lower genome-wide polygenic score was associated with a greater likelihood of suicidal ideation. The overall suicide risk associated with genome-wide polygenic scores was greatest for ADHD, with a significantly higher OR across all suicidal phenotypes among both participants in the multiethnic cohort and participants with European ancestry only. This association between the genome-wide polygenic score for ADHD and the risk of suicidal thoughts and behaviors was consistent with findings from previous studies,^51,62^ suggesting that ADHD was associated with suicide attempts among youths. Our genome-wide polygenic score results highlighted possible genetic overlap between ADHD and suicidal thoughts and behaviors among youths. The explained variance (as measured by McFadden pseudo-*R*^2^)^63^ of suicide attempts associated with the genome-wide polygenic score for ADHD was approximately 10.0% among all children in the multiethnic cohort and 10.5% among children with European ancestry only. This estimation was higher than the results from previous studies of suicidal thoughts and behaviors measured by polygenic scores,^64,65,66^ which reported a maximum variance explained by 0.13% to 0.20% of self-harm behaviors^64^ or up to 0.30% to 0.70% of the phenotypic variance for suicide attempt explained by the depression-based genome-wide polygenic score.^65,66^ When we repeated the analysis by restricting it to only healthy children without any KSADS-Comp diagnosis, the genome-wide polygenic score for PTSD appeared to have the most significant association with suicidal phenotypes among all participants in the multiethnic cohort and participants with European ancestry only.

The genome-wide polygenic score for ASD was not only associated with active suicidal ideation but also had a significant interaction with early life stress. These results were consistent with previous findings.^67,68^ One study reported that youths with ASD were 28 times more likely to have suicidal thoughts or behaviors than their peers without ASD.^69^ The significant association between autistic traits and the risk of suicidal thoughts and behaviors could be explained by behavioral attributes of both phenotypes, such as low socialization and problem-solving skills or increased levels of impulsivity and anxiety.^68^ Although the literature has reported several social risk factors underlying the association between ASD and suicidal thoughts and behaviors,^69,70^ the genetic and environmental factors associated with the overlap of ASD and suicidal behaviors remain unknown.^71^ Our analysis revealed that the association between the genetic risk of ASD and the risk of suicide differed significantly by adverse childhood experience. To our knowledge, this study is the first to report a gene-environment interaction underlying the association of ASD with suicidal thoughts and behaviors. These results suggest that, among those with a genetic predisposition to ASD, an adverse childhood experience may increase the risk of suicidal thoughts and behaviors.

Consistent with our findings, previous studies^51,62,72^ reported high suicide risk among individuals who had ADHD in addition to ASD. Given that 20% to 50% of individuals with ASD are known to have comorbid ADHD,^72^ the significant association of the genome-wide polygenic scores for ASD and ADHD with suicidal thoughts and behaviors suggests that a genetic predisposition to psychiatric comorbidity may be associated with high suicide risk among youths.

Our study findings suggested that a higher genetic predisposition to general happiness (ie, the belief that one’s life is meaningful) may be associated with a decreased risk of suicidal thoughts and behaviors among youths. Of the several subjective well-being measures (eg, happy in general or happy with one’s health in general), only the genome-wide polygenic score for the belief that one's life is meaningful was significantly associated with suicidal thoughts and behavior among youths. Previous studies have reported a negative association between subjective happiness and suicide.^37,38^

To our knowledge, the present study provided the first evidence of the utility of the genome-wide polygenic score for assessing risk of suicide among a pediatric population. The integration of multitrait genome-wide polygenic scores, family environmental factors, behavioral and psychological scales, and early life stress assessment allowed better estimation of overall suicidal thoughts and behaviors (AUROC of 0.77 among children with European ancestry only and 0.71 among all children in the multiethnic cohort) and overall suicidal ideation (AUROC of 0.76 among children with European ancestry only and 0.75 among all children in the multiethnic cohort) compared with the baseline (questionnaire-based) models. Possibly owing to the limited sample size, the assessment of risk of suicide attempt did not have reliable performance, even though the integrated model outperformed the baseline model that did not include any self-reported phenotype data (eTable 6 in the Supplement). Based on the estimated importance of input features, the behavioral and psychological scales (ie, the CBCL), including scales measuring depressive symptoms, anxiety and depression, internalizing symptoms, and externalizing symptoms, were most consistently associated with suicidal thoughts and behaviors across the models (eTable 7 in the Supplement). With regard to assessing risk of suicidal thoughts and behaviors, among all participants in the multiethnic cohort, the addition of genome-wide polygenic scores did not always increase the AUROC of the integrative model compared with that of the baseline model. This finding might suggest limited transferability of European GWAS-based polygenic scores to multiethnic individuals.

To our knowledge, no studies with similar data or approaches have been conducted among youths; however, previous studies of adults that examined sociodemographic and psychiatric risk factors reported similar classification performance, ranging from 0.74 to 0.88.^73,74,75,76^ However, some of those studies included longitudinal measurements of risk factors (eg, 12-month or lifelong risk factors) that may be inaccessible in clinical practice, unlike the genetic and cross-sectional questionnaires used in our study.

### Limitations

This study has several limitations. We used European ancestry–based GWASs to estimate genome-wide polygenic scores among ethnically diverse samples. The transferability of our findings across ethnic groups may be improved with the use of better methods to measure genome-wide polygenic scores among multiethnic individuals, which is currently an active area of research.^77^ In addition, because of the scarcity of completed suicides among preadolescent youths, we used suicidal ideation and suicide attempt as proxy phenotypes for suicidal behavior.^32,78^ It should be noted that not all suicidal ideation or suicide attempts lead to completed suicide. Although we used the KSADS-Comp–derived feature for the measurement of suicidal thoughts and behaviors to ensure generalizability, the definitions of suicidal thoughts and behaviors are heterogenous and without universal agreement. For estimating the risk of suicide attempt, the number of testing samples may be suboptimal for certain analyses (eg, AUROC). Nevertheless, given that the ABCD study is, to our knowledge, the largest long-term observational study of children’s health in the US to date, the data set used in the current study was the largest available for analysis. We calculated other performance measures, such as positive predictive values, negative predictive values, and accuracy, in an effort to better assess the reproducibility of the models.

## Conclusions

This cohort study found that multiple genome-wide polygenic scores were significantly associated with suicidal thoughts and behaviors among youths. These results highlight the importance and potential utility of the genome-wide polygenic score approach to early screening for high risk of suicidal behaviors among pediatric populations. The study’s findings may also motivate further development of genome-wide polygenic score–based screening methods and intervention strategies for youths at risk of suicide. With the integrative model developed in this study, suicide prevention programs could further tailor strategies to subgroups with distinct risk profiles, such as children with high polygenic scores for particular traits or children with high genetic vulnerability to early life stress, which may improve suicide intervention and prevention strategies for youths.

## Notes

Supplement. **eMethods.** Genotype Data and Outcomes and Measures **eResults.** Multiethnic Participants **eTable 1.** Genome-Wide Association Study List for Generating the Polygenic Scores of 24 Common and Psychiatric Traits **eTable 2.** Suicidal Ideation and Attempt Items in Detail **eTable 3.** Sociodemographic and Clinical Outcomes of Participants From the Adolescent Brain and Cognitive Development (ABCD) Study **eTable 4.** Top Results of Main Analysis of Association Between Multitrait Genome-Wide Polygenic Scores and Suicidal Phenotypes **eTable 5.** Top Results of Sensitivity Analysis Using Only Healthy Control Group Without Any Records From the Kiddie Schedule for Affective Disorders and Schizophrenia **eTable 6.** Prediction Performance of 3 Machine Learning Models Based on Genome-Wide Polygenic Scores and Cognitive, Psychological, Behavioral, Environmental, and Familial Variables for Suicidal Thoughts and Behaviors Among Youths **eTable 7.** Feature Importance of Elastic Net Model for Prediction of Suicidal Thoughts and Behaviors Among Youths **eTable 8.** Early Life Stress Scale in Detail **eFigure 1.** Biplot From Principal Component Analysis of the Combined Genotype Data of the 1000-Genome Reference Panel (Phase 3, Release 5) and Study Participants From the Adolescent Brain and Cognitive Development (ABCD) **eFigure 2.** Analysis of the Association Between Multitrait Genome-Wide Polygenic Scores and Suicidal Thoughts and Behaviors Among 6592 Children in the Multiethnic Cohort
eReferences
