# Supplementary material for: Association of Genome-Wide Polygenic Scores for Multiple Psychiatric and Common Traits in Preadolescent Youths at Risk of Suicide
Source: JAMA Netw Open. 2022 Feb 21;5(2):e2148585. doi: 10.1001/jamanetworkopen.2021.48585 (PMC8861848; doi:10.1001/jamanetworkopen.2021.48585)
Supplement: Supplement. — eMethods. Genotype Data and Outcomes and Measures eResults. Multiethnic Participants eTable 1. Genome-Wide Association Study List for Generating the Polygenic Scores of 24 Common and Psychiatric Traits eTable 2. Suicidal Ideation and Attempt Items in Detail eTable 3. Sociodemographic and Clinical Outcomes of Participants From the Adolescent Brain and Cognitive Development (ABCD) Study eTable 4. Top Results of Main Analysis of Association Between Multitrait Genome-Wide Polygenic Scores and Suicidal Phenotypes eTable 5. Top Results of Sensitivity Analysis Using Only Healthy Control Group Without Any Records From the Kiddie Schedule for Affective Disorders and Schizophrenia eTable 6. Prediction Performance of 3 Machine Learning Models Based on Genome-Wide Polygenic Scores and Cognitive, Psychological, Behavioral, Environmental, and Familial Variables for Suicidal Thoughts and Behaviors Among Youths eTable 7. Feature Importance of Elastic Net Model for Prediction of Suicidal Thoughts and Behaviors Among Youths eTable 8. Early Life Stress Scale in Detail eFigure 1. Biplot From Principal Component Analysis of the Combined Genotype Data of the 1000-Genome Reference Panel (Phase 3, Release 5) and Study Participants From the Adolescent Brain and Cognitive Development (ABCD) eFigure 2. Analysis of the Association Between Multitrait Genome-Wide Polygenic Scores and Suicidal Thoughts and Behaviors Among 6592 Children in the Multiethnic Cohort eReferences [file jamanetwopen-e2148585-s001.pdf]

Supplementary Online Content

Joo YY, Moon SY, Wang HH, et al. Association of genome-wide polygenic scores for multiple psychiatric and common traits in preadolescent youths at risk of suicide. *JAMA Netw Open*. 2022;5(2):e2148585. doi:10.1001/jamanetworkopen.2021.48585

eMethods. Genotype Data and Outcomes and Measures

eResults. Multiethnic Participants

eTable 1. Genome-Wide Association Study List for Generating the Polygenic Scores of 24 Common and Psychiatric Traits

eTable 2. Suicidal Ideation and Attempt Items in Detail

eTable 3. Sociodemographic and Clinical Outcomes of Participants From the Adolescent Brain and Cognitive Development (ABCD) Study

eTable 4. Top Results of Main Analysis of Association Between Multitrait Genome-Wide Polygenic Scores and Suicidal Phenotypes

eTable 5. Top Results of Sensitivity Analysis Using Only Healthy Control Group Without Any Records From the Kiddie Schedule for Affective Disorders and Schizophrenia

eTable 6. Prediction Performance of 3 Machine Learning Models Based on Genome-Wide Polygenic Scores and Cognitive, Psychological, Behavioral, Environmental, and Familial Variables for Suicidal Thoughts and Behavior Among Youths

eTable 7. Feature Importance of Elastic Net Model for Prediction of Suicidal Thoughts and Behaviors Among Youths

eTable 8. Early Life Stress Scale in Detail

eFigure 1. Biplot From Principal Component Analysis of the Combined Genotype Data of the 1000-Genome Reference Panel (Phase 3, Release 5) and Study Participants From the Adolescent Brain and Cognitive Development (ABCD)

eFigure 2. Analysis of the Association Between Multitrait Genome-Wide Polygenic Scores and Suicidal Thoughts and Behaviors Among 6592 Multiethnic Children

eReferences

This supplementary material has been provided by the authors to give readers additional information about their work.

© 2022 Joo YY et al. *JAMA Network Open*.

eMethods. Genotype Data and Outcomes and Measures

Genotype data

Our study samples have complex genetic structures owing to the presence of numerous familial relatives, diverse genetic ancestries and admixed samples innate to the U.S. population. Identifying sample relatedness in the presence of genetic-ancestry variation and vice versa is challenging especially in admixed populations, thus robust approaches for identifying each of these structures were implemented in this study. We used PC-Air to estimate ancestrally-informative principal components(PCs) of the genotypes that is robust to the related pedigree structure, and PC-Relate to provide accurate estimate of recent genetic relatedness measures from the population with ancestry admixture.

In this study, we estimated both kinship coefficients (KCs) and principal components (PCs) to control familial relatedness, population structures and ancestry admixture by using both PC-Air [1] and PC-Relate [2] Firstly, we selected autosomal SNPs by LD pruning such that all pairs had $r^2 < 0.1$ in a sliding 10Mb window in a set of individuals estimated to be more distant than 3rd degree relatives. This selection procedure resulted in approximately 718,125 SNPs with missing call rate < 5% and MAF > 0.5%. Then, we obtained initial KC estimates with KING-robust, which is robust to discrete population structure but is known to be relatively biased in admixed individuals. Then we performed the PC analysis with PC-Air, which is robust to the genetic relatedness identified in the sample. Based on the computed PCs, we found updated KC estimates with PC-Relate, which accounts for population structure and provides accurate estimates in the presence of admixture. At this point, we plotted the genotype PCs of the unrelated samples and determined that the 5th and 6th PC mainly separated 228 outliers with high proportions of East Asian or African ancestry from the remaining subjects. (**eFigure 1**). Since these individuals seems to be genetically distinct from the rest of the individuals, we removed the outliers fell outside the 6 standard deviation limits from the center in PC5-6 space, and repeated the PC-Air and PC-Relate steps described above. To prevent the potential misidentification, we performed the steps until there were no extreme PC outliers in the final sample set and all pairs of the selected individuals inferred to be 4th degree relatives or farther (KC > 0.022).

Outcomes and Measures

The following variables were additionally considered for inputs to the classification models. For psychopathology, ABCD Parent-Child Behavior Checklist (CBCL); for intelligence, NIH toolbox;58 for a family environment, Youth Family Environment Scale; and for ELS, abuse (physical and sexual), household challenges (family substance abuse, family mental illness, family criminals, parental separation, or divorce, and violently treated mothers) and neglect (emotional and physical neglect). The items assessing ELS were extracted from various measurement tools reported by children themselves or parents (**eTable 8**). An ELS composite score was calculated by averaging z-standardized scores of each subtype. Higher A higher composite score indicates a more severe ELS.

© 2022 Joo YY et al. *JAMA Network Open*.

eResults. Multiethnic Participants

In the entire, multiethnic participants, the results largely remained unchanged for predicting suicidal ideation. The ROC-AUC of the integrative prediction model increased to 0.746 (95% CI, 0.708-784; Accuracy, 0.670), whereas the ROC-AUC of the baseline model was 0.584 (95% CI, 0.550-0.618; Accuracy, 0.529). However, we did not detect significant improvement in predicting overall suicidality and suicide attempts with multitrait GPSs. The estimated feature importance showed that the top 10 important features for prediction were mostly CBCL measures, such as assessing depressive symptoms or internalizing symptoms, except for ADHD GPS for overall suicidality prediction (**eTable 7B**). Without self-reported questionnaire data, the baseline model showed a ROC-AUC of 0.550 (95% CI, 0.517-0.582; Accuracy,0.545) for overall suicidality and 0.652 (95% CI, 0.559-0.746; Accuracy, 0.536) for suicide attempt. In the presence of GPS and self-reported phenotypes, the ROC-AUC increased to 0.705 (95% CI, 0.665-0.746; Accuracy, 0.682) for overall suicidality and 0.725 (95% CI, 0.571-0.878; Accuracy, 0.765) for suicide attempt. All prediction results are based on the elastic net model, and the results for logistic regression and random forest classifier are presented in **eTable 6B.**

© 2022 Joo YY et al. *JAMA Network Open*.

eTable 1. Genome-Wide Association Study List for Generating the Polygenic Scores of 24 Common and Psychiatric Traits

Trait	Original GWAS Sample Size	Download link for Summary Statistics	Study
**Attention-deficit/hyperactivity disorder (ADHD)**	55,374	https://www.med.unc.edu/pgc/download-results/adhd/?choice=Attention+Deficit+Hyperactivity+Disorder+%28ADHD%29	Demontis, D., Walters, R.K., Martin, J. et al. (2019). Discovery of the first genome-wide significant risk loci for attention deficit/hyperactivity disorder. Nature Genetics. 51, 63-75. https://doi.org/10.1038/s41588-018-0269-7
**Autism spectrum disorder (ASD)**	46,350	https://www.med.unc.edu/pgc/results-and-downloads/	Grove, J., Ripke, S., Als, T.D. et al. (2019). Identification of common genetic risk variants for autism spectrum disorder. Nature Genetics. 51, 431-444. https://doi.org/10.1038/s41588-019-0344-8
**Bipolar disorder (BIP)**	51,710	https://www.med.unc.edu/pgc/results-and-downloads	Stahl, E.A., Breen, G., Forstner, A.J. et al. (2019). Genome-wide association study identifies 30 loci associated with bipolar disorder. Nature Genetics. 51, 793-803. https://doi.org/10.1038/s41588-019-0397-8
**Schizophrenia (SCZ)**	65,967	https://www.med.unc.edu/pgc/download-results/scz-bip/?choice=Schizophrenia+%28SCZ%29Schizophrenia+%28SCZ%29+%2B+Bipolar+Disorder+%28BIP%29	Douglas M. Ruderfer (2018). Genomic Dissection of Bipolar Disorder and Schizophrenia, Including 28 Subphenotypes. Cell. 173(7), 1705-1715. https://doi.org/10.1016/j.cell.2018.05.046
**Major depressive disorder (MDD)**	807,553	https://doi.org/10.7488/ds/2458	Howard, D.M., Adams, M.J., Clarke, T. et al. (2019). Genome-wide meta-analysis of depression identifies 102 independent variants and highlights the importance of the prefrontal brain regions. Nature Neuroscience. 22. 343-352. https://doi.org/10.1038/s41593-018-0326-7
**Posttraumatic stress disorder (PTSD)**	206,655	https://www.med.unc.edu/pgc/results-and-downloads	Nievergelt, C.M., Maihofer, A.X., Klengel, T. et al. (2019). International meta-analysis of PTSD genome-wide association studies identifies sex - and ancestry - specific genetic risk loci. Nature Communications. 10, 4558.

© 2022 Joo YY et al. *JAMA Network Open*.

			https://doi.org/10.1038/s41467-019-12576-w
**Substance use disorders (SUD)**	184,765	http://ldsc.broadinstitute.org/gwashare/	Pasman, J.A., Verweij, K.J.H., Gerring, Z. et al. (2018). GWAS of lifetime cannabis use reveals new risk loci, genetic overlap with psychiatric traits, and a causal effect of schizophrenia liability. Nature Neuroscience. 21, 1161-1170. https://doi.org/10.1038/s41593-018-0206-1
**IQ**	269,867	https://ctg.cncr.nl/software/summary_statistics	Savage JE, et al. Genome-wiide association meta-analysis (N=269,867) identifies new genetic and functional links to intelligence. Nature Genetics, 2018 Jul;50(7):912-919
**Depression**	381,455	https://ctg.cncr.nl/software/summary_statistics	Meta-analysis of genome-wide association studies for neuroticism in 449,484 individuals identifies novel genetic loci and pathways (Nagel et al., Nature Genetics, 2018)
**Worry subcluster**	348,219	https://ctg.cncr.nl/software/summary_statistics	Meta-analysis of genome-wide association studies for neuroticism in 449,484 individuals identifies novel genetic loci and pathways (Nagel et al., Nature Genetics, 2018)
**Alzheimer disease**	455,258	https://ctg.cncr.nl/software/summary_statistics	Genome-wide meta-analysis identifies new loci and functional pathways influencing Alzheimer's disease risk Nature Genetics, 2019 Mar;51(3):404-413. doi: 10.1038/s41588-018-0311-9
**insomnia**	386,533	https://ctg.cncr.nl/software/summary_statistics	Genome-wide analysis of insomnia in 1,331,010 individuals identifies new risk loci and functional pathways . Nature Genetics 2019 Mar;51(3):394-403. doi: 10.1038/s41588-018-0333-3.
**Snoring**	359,916	https://ctg.cncr.nl/software/summary_statistics	Genome-wide analysis of insomnia in 1,331,010 individuals identifies new risk loci and functional pathways . Nature Genetics 2019 Mar;51(3):394-403. doi: 10.1038/s41588-018-0333-3.

© 2022 Joo YY et al. *JAMA Network Open*.

**General risk tolerance**	466,571	https://www.thessgac.org/data	Karlsson LinnÃr et al. (2019). Genome-wide association analyses of risk tolerance and risky behaviors in over one million individuals identify hundreds of loci and shared genetic influences. Nature Genetics 51; 245-257.
**Drinks per week**	414,343	https://www.thessgac.org/data	Karlsson LinnÃr et al. (2019). Genome-wide association analyses of risk tolerance and risky behaviors in over one million individuals identify hundreds of loci and shared genetic influences. Nature Genetics 51; 245-257.
**Smoking status (ever smoker)**	518,633	https://www.thessgac.org/data	Karlsson LinnÃr et al. (2019). Genome-wide association analyses of risk tolerance and risky behaviors in over one million individuals identify hundreds of loci and shared genetic influences. Nature Genetics 51; 245-257.
**The first PC of the four risky behaviors**	315,894	https://www.thessgac.org/data	Karlsson LinnÃr et al. (2019). Genome-wide association analyses of risk tolerance and risky behaviors in over one million individuals identify hundreds of loci and shared genetic influences. Nature Genetics 51; 245-257.
**Happiness and subjective well-being - general happiness**	126,132	http://www.nealelab.is/uk-biobank/	UK Biobank GWAS. Neale Lab. http://www.nealelab.is/uk-biobank/ Accessed Apr 29. 2020.
**Happiness and subjective well-being - general happiness with own health**	126,477	http://www.nealelab.is/uk-biobank/	UK Biobank GWAS. Neale Lab. http://www.nealelab.is/uk-biobank/ Accessed Apr 29. 2020.
**Happiness and subjective well-being - belief that own life is meaningful**	123,818	http://www.nealelab.is/uk-biobank/	UK Biobank GWAS. Neale Lab. http://www.nealelab.is/uk-biobank/ Accessed Apr 29. 2020.
**Subjective well-being**	128,677	http://www.thessgac.org/#!data/kuzq8	Okbay, A., Baselmans, B., De Neve, JE. et al. Genetic variants associated with subjective well-being, depressive symptoms, and neuroticism identified through genome-wide analyses. Nat Genet 48, 624–633 (2016). https://doi.org/10.1038/ng.3552
**Educational attainment**	766,345	http://www.thessgac.org/data	Lee et al. (2018). Gene discovery and polygenic prediction from a 1.1-million-person GWAS of educational attainment. Nature Genetics, 50(8), 1112-1121. doi: 10.1038/s41588-018-0147-3

© 2022 Joo YY et al. *JAMA Network Open*.

**Cognitive performance**	257,828	http://www.thessgac.org/data	Lee et al. (2018). Gene discovery and polygenic prediction from a 1.1-million-person GWAS of educational attainment. Nature Genetics, 50(8), 1112-1121. doi: 10.1038/s41588-018-0147-3
**Automobile speeding propensity**	404,291	https://www.thessgac.org/data	Karlsson LinnÃr et al. (2019). Genome-wide association analyses of risk tolerance and risky behaviors in over one million individuals identify hundreds of loci and shared genetic influences. Nature Genetics 51; 245-257.

© 2022 Joo YY et al. *JAMA Network Open*.

eTable 2. Suicidal Ideation and Attempt Items in Detail

Suicidality features	Sub-scale	K-SADS items		Item description
		Current	Past	
Passive suicidal ideation	Passive suicidal ideation	ksads_23_946_t	ksads_23_957_t	A current or previous wish to be dead or thought to be better off dead
		ksads_23_946_p	ksads_23_957_p	
Active suicidal ideation	Active non-specific suicidal ideation	ksads_23_947_t	ksads_23_958_t	A current or previous desire to kill oneself or die by suicide
		ksads_23_947_p	ksads_23_958_p	
	Active suicidal ideation with a specific method	ksads_23_948_t	ksads_23_959_t	A current or previous thought about how to die by suicide
		ksads_23_948_p	ksads_23_959_p	
	Active suicidal ideation with intent	ksads_23_949_t	ksads_23_960_t	A current or previous intent to act on suicidal thought
		ksads_23_949_p	ksads_23_960_p	
	Active suicidal ideation with plan	ksads_23_950_t	ksads_23_961_t	A current or previous thought about specific plan on suicide
		ksads_23_950_p	ksads_23_961_p	
Suicidal attempt	Suicidal attempt	ksads_23_954_t	ksads_23_965_t	Lifetime experience of suicidal attempt
		ksads_23_954_p	ksads_23_965_p	

© 2022 Joo YY et al. *JAMA Network Open*.

eTable 3. Sociodemographic and Clinical Outcomes of Participants From the Adolescent Brain and Cognitive Development (ABCD) Study

(A) The entire multiethnic participants used for the prediction task, imputed for continous variables (n=7,140)

	**Youth with suicidality (n=1,012)**		**Control Youth (N=6,128)**		
	**Count or Mean**	**Percentage (%) or SD**	**Count or Mean**	**Percentage (%) or SD**	**Statistics**
**Female**	618	*61.1%*	2,970	*48.5%*	$c^2 = 31.3$ (P < .001)
**Mean age (in months)**	118.7	*7.35*	119	*7.31*	t = -1.08 (P = .28)
**Mean income (bracket in 1~10 scales)**	7.1	*2.27*	7.42	*2.22*	t = -4.19 (P < .001)
**Marital status of the family (currently married)**	631	*62.4%*	4,360	*71.1%*	$c^2 = 41.2$ (P < .001)
**Mean mother's educational attainment**	16.7	*2.57*	16.7	*2.72*	t = -0.65 (P = .52)
**Self-reported race (white)**	547	*54.1%*	3,438	*56.1%*	$c^2 = 11.5$ (P = .02)
**Site for sample collection (site ID of largest sample = 16)**	94	*9.29%*	560	*9.14%*	$c^2 = 33.5$ (P = .04)

**The scale of mean income is as follows. 1= Less than $5,000; 2=$5,000 to $11,999; 3=$12,000 to $15,999; 4=$16,000 to $24,999; 5=$25,000 to $34,999; 6=$35,000 to $49,999; 7=$50,000 to $74,999; 8= $75,000 to $99,999; 9=$100,000 to $199,999; 10=$200,000 or greater. through $74,999; 8= $75,000 through $99,999; 9=$100,000 through $199,999; 10=$200,000 and greater.*

© 2022 Joo YY et al. *JAMA Network Open*.

(B) The European-ancestry participants used for ancestry-stratified analysis (n=5,888)

	**Youth with suicidality (n=826)**		**Control Youth (N=5,062)**		
	**Count or Mean**	**Percentage (%) or SD**	**Count or Mean**	**Percentage (%) or SD**	**Statistics**
**Female**	523	*63.3%*	2,684	*53.00%*	c^2 = 27.1 (P < .001)
**Mean age (in months)**	118.8	*7.42*	119.0	*7.34*	t = -0.76 (P = . 45)
**Mean income (bracket in 1~10 scales)**	7.44	*2.11*	7.84	*1.96*	t = -4.83 (P < .001)
**Marital status of the family (currently married)**	565	*68.4%*	3,926	*77.6%*	c^2 = 43.4 (P < .001)
**Mean mother's educational Attainment**	17	*2.3*	17.2	*2.37*	t = -1.98 (P = .048)
**Self-reported race (white)**	569	*68.9%*	3,515	*69.4%*	c^2 = 7.45 (P = .11)
**Site for sample collection (site ID of largest sample = 16)**	117	*14.2%*	638	*12.6%*	c^2 = 34.9 (P = .03)
**The scale of mean income is as follows. 1= Less than $5,000; 2=$5,000 to $11,999; 3=$12,000 to $15,999; 4=$16,000 to $24,999; 5=$25,000 to $34,999; 6=$35,000 to $49,999; 7=$50,000 to $74,999; 8= $75,000 to $99,999; 9=$100,000 to $199,999; 10=$200,000 or greater. through $74,999; 8= $75,000 through $99,999; 9=$100,000 through $199,999; 10=$200,000 and greater.*					

© 2022 Joo YY et al. *JAMA Network Open*.

eTable 4. Top Results of Main Analysis of Association Between Multitrait Genome-Wide Polygenic Scores and Suicidal Phenotypes (*P* < .05)

(A) Analysis of entire multiethnic participants (n=6,587)

Outcome	GPS predictor	Beta	SE	OR	p-value	Total N	N of cases	FDR significance
**Outcome**	GPS predictor	**Beta**	**SE**	**OR**	**p-value**	**Total N**	**N of cases**	**FDR significance**
Suicidalideation	ADHD	0.12	0.04	1.12	1.34.E-03	6587	930	TRUE
SuicidalideationActive	ADHD	0.16	0.05	1.17	1.43.E-03	6146	489	TRUE
SuicideAttempt	SCZ	0.41	0.13	1.50	1.60.E-03	5721	64	TRUE
Suicide_all	ADHD	0.11	0.04	1.12	1.69.E-03	6592	935	TRUE
Suicidalideation	GENERALHAPPINESS_MEANINGFUL	-0.11	0.04	0.89	1.78.E-03	6587	930	TRUE
Suicide_all	GENERALHAPPINESS_MEANINGFUL	-0.11	0.04	0.90	2.88.E-03	6592	935	FALSE
SuicidalideationActive	ASD	0.14	0.05	1.15	3.21.E-03	6146	489	FALSE
SuicidalideationPassive	ADHD	0.12	0.04	1.12	3.37.E-03	6431	774	FALSE
SuicidalideationActive	INSOMNIA	0.14	0.05	1.15	4.36.E-03	6146	489	FALSE
Suicide_all	PTSD	0.10	0.04	1.10	5.19.E-03	6592	935	FALSE
Suicidalideation	PTSD	0.10	0.04	1.10	5.83.E-03	6587	930	FALSE
SuicidalideationPassive	PTSD	0.11	0.04	1.11	5.89.E-03	6431	774	FALSE
SuicidalideationPassive	GENERALHAPPINESS_MEANINGFUL	-0.11	0.04	0.90	5.92.E-03	6431	774	FALSE
SuicideAttempt	GENERALHAPPINESS	-0.37	0.13	0.69	6.05.E-03	5721	64	FALSE
Suicidalideation	MDD	0.10	0.04	1.10	6.64.E-03	6587	930	FALSE
Suicide_all	MDD	0.10	0.04	1.10	7.31.E-03	6592	935	FALSE
SuicidalideationPassive	MDD	0.10	0.04	1.10	1.04.E-02	6431	774	FALSE
Suicidalideation	INSOMNIA	0.09	0.04	1.09	1.22.E-02	6587	930	FALSE
Suicide_all	INSOMNIA	0.09	0.04	1.09	1.23.E-02	6592	935	FALSE
SuicideAttempt	IQ	0.32	0.13	1.37	1.48.E-02	5721	64	FALSE
SuicidalideationPassive	SMOKER	0.09	0.04	1.09	2.49.E-02	6431	774	FALSE
Suicidalideation	ASD	0.08	0.04	1.08	2.75.E-02	6587	930	FALSE
Suicidalideation	SMOKER	0.08	0.04	1.08	3.55.E-02	6587	930	FALSE
Suicide_all	SMOKER	0.07	0.04	1.08	3.88.E-02	6592	935	FALSE
Suicide_all	ASD	0.07	0.04	1.08	3.88.E-02	6592	935	FALSE

© 2022 Joo YY et al. *JAMA Network Open*.

(B) Analysis of European-ancestry participants (n=5,374)

Outcome	GPS predictor	Beta	SE	OR	p-value	Total N	N of cases	FDR significance
Suicidalideation	ADHD	0.13	0.04	1.14	8.70.E-04	5372	735	TRUE
Suicide_all	ADHD	0.13	0.04	1.14	1.02.E-03	5374	737	TRUE
SuicidalideationPassive	ADHD	0.14	0.04	1.15	1.27.E-03	5257	620	TRUE
SuicideAttempt	SCZ	0.48	0.15	1.61	1.27.E-03	4682	45	TRUE
SuicidalideationActive	ADHD	0.17	0.05	1.19	1.42.E-03	5011	374	TRUE
SuicidalideationActive	ASD	0.17	0.05	1.18	1.70.E-03	5011	374	TRUE
Suicide_all	MDD	0.12	0.04	1.12	2.67.E-03	5374	737	TRUE
Suicidalideation	MDD	0.12	0.04	1.12	2.71.E-03	5372	735	TRUE
Suicidalideation	GENERALHAPPINESS_MEANINGFUL	-0.12	0.04	0.89	2.80.E-03	5372	735	TRUE
Suicide_all	GENERALHAPPINESS_MEANINGFUL	-0.12	0.04	0.89	3.04.E-03	5374	737	TRUE
Suicide_all	PTSD	0.11	0.04	1.12	3.87.E-03	5374	737	TRUE
Suicidalideation	PTSD	0.11	0.04	1.12	4.04.E-03	5372	735	TRUE
SuicidalideationPassive	GENERALHAPPINESS_MEANINGFUL	-0.12	0.04	0.89	4.58.E-03	5257	620	TRUE
SuicidalideationPassive	MDD	0.12	0.04	1.12	5.50.E-03	5257	620	TRUE
SuicidalideationPassive	PTSD	0.12	0.04	1.12	5.52.E-03	5257	620	TRUE
Suicide_all	SMOKER	0.10	0.04	1.11	9.52.E-03	5374	737	FALSE
Suicidalideation	SMOKER	0.10	0.04	1.11	9.68.E-03	5372	735	FALSE
SuicidalideationPassive	RISK4PC	0.10	0.04	1.11	1.37.E-02	5257	620	FALSE
SuicidalideationPassive	SMOKER	0.10	0.04	1.11	1.94.E-02	5257	620	FALSE
SuicideAttempt	IQ	0.34	0.15	1.41	2.62.E-02	4682	45	FALSE
Suicidalideation	INSOMNIA	0.09	0.04	1.09	2.88.E-02	5372	735	FALSE
Suicide_all	INSOMNIA	0.09	0.04	1.09	3.17.E-02	5374	737	FALSE
Suicide_all	RISK4PC	0.08	0.04	1.09	3.31.E-02	5374	737	FALSE
Suicidalideation	RISK4PC	0.08	0.04	1.09	3.63.E-02	5372	735	FALSE
SuicidalideationActive	NEUROTICISM	-0.11	0.05	0.89	3.70.E-02	5011	374	FALSE

© 2022 Joo YY et al. *JAMA Network Open*.

eTable 5. Top Results of Sensitivity Analysis Using Only Healthy Control Group Without Any KSADS Records (*P* < .05)

(A) Analysis of entire multiethnic participants (n=2,587)

Outcome	GPS predictor	OR	P-value	N of cases	Bonferroni significance
Suicide_all	PTSD	1.17 (1.07 - 1.27)	3.35.E-04	935	TRUE
Suicidalideation	PTSD	1.16 (1.07 - 1.27)	3.90.E-04	930	TRUE
SuicidalideationPassive	PTSD	1.17 (1.07 - 1.28)	5.44.E-04	774	TRUE
SuicidalideationActive	ASD	1.20 (1.08 - 1.33)	9.50.E-04	489	TRUE
SuicideAttempt	SCZ	1.53 (1.17 - 1.99)	2.05.E-03	64	TRUE
SuicidalideationActive	ADHD	1.19 (1.06 - 1.33)	2.47.E-03	489	TRUE
Suicidalideation	ADHD	1.13 (1.04 - 1.23)	4.52.E-03	930	FALSE
Suicide_all	ADHD	1.13 (1.04 - 1.23)	5.22.E-03	935	FALSE
Suicidalideation	GENERALHAPPINESS_MEANINGFUL	0.89 (0.81 - 0.97)	5.84.E-03	930	FALSE
SuicidalideationPassive	SMOKER	1.13 (1.04 - 1.24)	5.85.E-03	774	FALSE
SuicidalideationActive	PTSD	1.16 (1.04 - 1.29)	8.31.E-03	489	FALSE
Suicide_all	GENERALHAPPINESS_MEANINGFUL	0.89 (0.82 - 0.97)	8.50.E-03	935	FALSE
SuicidalideationPassive	ADHD	1.13 (1.03 - 1.24)	8.60.E-03	774	FALSE
Suicidalideation	SMOKER	1.12 (1.03 - 1.22)	8.64.E-03	930	FALSE
Suicide_all	SMOKER	1.12 (1.03 - 1.22)	9.16.E-03	935	FALSE
Suicidalideation	MDD	1.12 (1.03 - 1.22)	9.70.E-03	930	FALSE
Suicide_all	MDD	1.12 (1.03 - 1.22)	1.05.E-02	935	FALSE
SuicidalideationActive	INSOMNIA	1.15 (1.03 - 1.28)	1.07.E-02	489	FALSE
SuicidalideationPassive	MDD	1.13 (1.03 - 1.23)	1.14.E-02	774	FALSE
Suicidalideation	ASD	1.11 (1.02 - 1.21)	1.32.E-02	930	FALSE
SuicideAttempt	GENERALHAPPINESS	0.70 (0.53 - 0.93)	1.34.E-02	64	FALSE
SuicidalideationPassive	GENERALHAPPINESS_MEANINGFUL	0.89 (0.82 - 0.98)	1.35.E-02	774	FALSE
Suicide_all	ASD	1.11 (1.02 - 1.20)	1.80.E-02	935	FALSE
SuicidalideationActive	SMOKER	1.13 (1.01 - 1.26)	2.93.E-02	489	FALSE
SuicideAttempt	IQ	1.35 (1.02 - 1.79)	3.41.E-02	64	FALSE
Suicidalideation	INSOMNIA	1.09 (1.01 - 1.19)	3.57.E-02	930	FALSE
Suicide_all	INSOMNIA	1.09 (1.01 - 1.19)	3.69.E-02	935	FALSE
SuicidalideationPassive	ASD	1.10 (1.00 - 1.20)	4.06.E-02	774	FALSE
SuicidalideationActive	MDD	1.12 (1.00 - 1.25)	4.87.E-02	489	FALSE
SuicidalideationPassive	EA	0.91 (0.83 - 1.00)	5.00.E-02	774	FALSE

© 2022 Joo YY et al. *JAMA Network Open*.

(B) Analysis of European-ancestry participants (n=2,088)

Outcome	GPS predictor	OR	P-value	N of cases	Bonferroni significance
Suicide_all	PTSD	1.18 (1.08 - 1.29)	3.03.E-04	737	TRUE
Suicidalideation	PTSD	1.18 (1.08 - 1.29)	3.17.E-04	735	TRUE
SuicidalideationPassive	PTSD	1.19 (1.08 - 1.30)	5.10.E-04	620	TRUE
Suicide_all	SMOKER	1.17 (1.06 - 1.29)	1.11.E-03	737	TRUE
Suicidalideation	SMOKER	1.17 (1.06 - 1.29)	1.13.E-03	735	TRUE
SuicidalideationActive	ASD	1.21 (1.08 - 1.37)	1.41.E-03	374	TRUE
SuicidalideationActive	ADHD	1.23 (1.08 - 1.39)	1.49.E-03	374	TRUE
SuicideAttempt	SCZ	1.62 (1.19 - 2.22)	2.36.E-03	45	TRUE
Suicidalideation	ADHD	1.16 (1.05 - 1.27)	2.42.E-03	735	TRUE
Suicide_all	ADHD	1.16 (1.05 - 1.27)	2.54.E-03	737	TRUE
SuicidalideationPassive	SMOKER	1.16 (1.05 - 1.29)	2.67.E-03	620	TRUE
SuicidalideationPassive	ADHD	1.16 (1.05 - 1.29)	2.94.E-03	620	TRUE
Suicide_all	MDD	1.14 (1.04 - 1.26)	5.58.E-03	737	TRUE
Suicidalideation	MDD	1.14 (1.04 - 1.26)	5.70.E-03	735	TRUE
SuicidalideationPassive	MDD	1.14 (1.03 - 1.26)	9.05.E-03	620	FALSE
SuicidalideationActive	SMOKER	1.17 (1.04 - 1.33)	9.81.E-03	374	FALSE
Suicidalideation	GENERALHAPPINESS_MEANINGFUL	0.89 (0.81 - 0.97)	9.86.E-03	735	FALSE
Suicide_all	GENERALHAPPINESS_MEANINGFUL	0.89 (0.81 - 0.97)	1.01.E-02	737	FALSE
SuicidalideationActive	PTSD	1.16 (1.03 - 1.30)	1.26.E-02	374	FALSE
SuicidalideationPassive	GENERALHAPPINESS_MEANINGFUL	0.88 (0.80 - 0.97)	1.26.E-02	620	FALSE
SuicidalideationPassive	EA	0.88 (0.79 - 0.98)	2.31.E-02	620	FALSE
SuicidalideationPassive	RISK4PC	1.12 (1.02 - 1.23)	2.36.E-02	620	FALSE
SuicidalideationActive	INSOMNIA	1.14 (1.01 - 1.29)	3.22.E-02	374	FALSE
Suicidalideation	INSOMNIA	1.10 (1.01 - 1.21)	3.75.E-02	735	FALSE
Suicide_all	INSOMNIA	1.10 (1.00 - 1.21)	4.01.E-02	737	FALSE

© 2022 Joo YY et al. *JAMA Network Open*.

eTable 6. Prediction Performance of 3 Machine Learning Models Based on Genome-Wide Polygenic Scores and Cognitive, Psychological, Behavioral, Environmental, and Familial Variables for Suicidal Thoughts and Behavior Among Youths

(A) Elastic net model

		European-ancestry individuals only						
	**Input Data**	**ROC**	**95% CI**	**Accuracy**	**Sensitivity**	**Specificity**	**PPV**	**NPV**
**Overall Suicidality**	Demographic + GPS + Environmental factors	**0.766**	0.727 - 0.805	0.672	0.764	0.580	0.645	0.711
	Demographic + Environmental factors	**0.730**	0.686 - 0.775	0.682	0.723	0.641	0.668	0.698
	Demographic + GPS	**0.575**	0.527 - 0.623	0.558	0.574	0.542	0.556	0.560
	Demographic only	**0.558**	0.519 - 0.598	0.557	0.510	0.603	0.562	0.552
**Suicidal Ideation**	Demographic + GPS + Environmental factors	**0.759**	0.718 - 0.800	0.660	0.739	0.581	0.638	0.690
	Demographic + Environmental factors	**0.731**	0.688 - 0.774	0.689	0.758	0.620	0.666	0.719
	Demographic + GPS	**0.603**	0.556 - 0.650	0.558	0.583	0.533	0.555	0.561
	Demographic only	**0.577**	0.539 - 0.616	0.544	0.511	0.577	0.547	0.542
**Suicide Attempt**	Demographic + GPS + Environmental factors	**0.929**	0.874 - 0.985	0.838	0.858	0.818	0.825	0.852
	Demographic + Environmental factors	**0.742**	0.564 - 0.920	0.681	0.803	0.557	0.645	0.739
	Demographic + GPS	**0.619**	0.456 - 0.781	0.673	0.694	0.651	0.665	0.680
	Demographic only	**0.636**	0.531 - 0.740	0.603	0.661	0.544	0.592	0.616

© 2022 Joo YY et al. *JAMA Network Open*.

		All individuals						
	**Input Data**	**ROC**	**95% CI**	**Accuracy**	**Sensitivity**	**Specificity**	**PPV**	**NPV**
**Overall Suicidality**	Demographic + GPS + Environmental factors	**0.705**	0.665 - 0.746	0.682	0.755	0.609	0.659	0.713
	Demographic + Environmental factors	**0.705**	0.664 - 0.746	0.665	0.726	0.603	0.647	0.688
	Demographic + GPS	**0.583**	0.542 - 0.624	0.547	0.580	0.514	0.544	0.551
	Demographic only	**0.550**	0.517 - 0.582	0.545	0.513	0.577	0.548	0.542
**Suicidal Ideation**	Demographic + GPS + Environmental factors	**0.746**	0.708 - 0.784	0.670	0.753	0.587	0.646	0.704
	Demographic + Environmental factors	**0.729**	0.691 - 0.767	0.663	0.725	0.600	0.645	0.686
	Demographic + GPS	**0.587**	0.546 - 0.629	0.553	0.561	0.545	0.552	0.553
	Demographic only	**0.584**	0.550 - 0.618	0.529	0.485	0.572	0.532	0.527
**Suicide Attempt**	Demographic + GPS + Environmental factors	**0.725**	0.571 - 0.878	0.765	0.834	0.697	0.733	0.807
	Demographic + Environmental factors	**0.736**	0.575 - 0.898	0.754	0.819	0.687	0.723	0.792
	Demographic + GPS	**0.661**	0.543 - 0.778	0.651	0.699	0.604	0.638	0.667
	Demographic only	**0.652**	0.559 - 0.746	0.536	0.702	0.371	0.527	0.555

© 2022 Joo YY et al. *JAMA Network Open*.

(B) Logistic regression (glm) and random forest model

			European-ancestry individuals only					
	Input Data	Method	ROC	95% CI	Sensitivity	Specificity	**PPV**	**NPV**
**Overall Suicidality**	Demographic + GPS + Environmental factors	GLM	**0.766**	0.724 - 0.807	0.707	0.619	0.650	0.679
		RandomForest	**0.755**	0.716 - 0.794	0.741	0.614	0.657	0.703
	Demographic + Environmental factors	GLM	**0.731**	0.687 - 0.776	0.723	0.646	0.671	0.700
		RandomForest	**0.723**	0.679 - 0.766	0.724	0.636	0.665	0.697
	Demographic + GPS	GLM	**0.556**	0.507 - 0.605	0.571	0.532	0.550	0.554
		RandomForest	**0.576**	0.528 - 0.624	0.576	0.527	0.549	0.554
	Demographic only	GLM	**0.558**	0.518 - 0.598	0.517	0.599	0.563	0.554
		RandomForest	**0.562**	0.522 - 0.602	0.517	0.589	0.557	0.549
**Suicidal Ideation**	Demographic + GPS + Environmental factors	GLM	**0.736**	0.693 - 0.780	0.704	0.625	0.652	0.679
		RandomForest	**0.758**	0.718 - 0.799	0.695	0.630	0.653	0.674
	Demographic + Environmental factors	GLM	**0.728**	0.684 - 0.772	0.721	0.646	0.671	0.698
		RandomForest	**0.724**	0.681 - 0.767	0.722	0.652	0.675	0.701
	Demographic + GPS	GLM	**0.591**	0.544 - 0.638	0.565	0.535	0.549	0.552
		RandomForest	**0.591**	0.544 - 0.637	0.545	0.534	0.539	0.540
	Demographic only	GLM	**0.578**	0.540 - 0.617	0.519	0.575	0.550	0.545
		RandomForest	**0.578**	0.538 - 0.617	0.501	0.592	0.551	0.543
**Suicide Attempt**	Demographic + GPS + Environmental factors	GLM	**0.718**	0.575 - 0.862	0.494	0.485	0.490	0.489
		RandomForest	**0.946**	0.902 - 0.990	0.858	0.745	0.771	0.840
	Demographic + Environmental factors	GLM	**0.446**	0.290 - 0.602	0.638	0.607	0.619	0.626
		RandomForest	**0.774**	0.632 - 0.917	0.782	0.599	0.661	0.733
	Demographic + GPS	GLM	**0.606**	0.438 - 0.773	0.612	0.630	0.623	0.619
		RandomForest	**0.756**	0.619 - 0.893	0.597	0.579	0.586	0.590

© 2022 Joo YY et al. *JAMA Network Open*.

	Demographic only	GLM	**0.633**	0.527 - 0.738	0.678	0.566	0.610	0.637
		RandomForest	**0.575**	0.460 - 0.689	0.503	0.558	0.532	0.529
**Overall Suicidality**	Demographic + GPS + Environmental factors	GLM	**0.766**	0.724 - 0.807	0.707	0.619	0.650	0.679
			All individuals					
	Input Data	Method	ROC	95% CI	Sensitivity	Specificity	**PPV**	**NPV**
**Overall Suicidality**	Demographic + GPS + Environmental factors	GLM	**0.706**	0.664 - 0.747	0.724	0.630	0.662	0.695
		RandomForest	**0.705**	0.666 - 0.743	0.739	0.603	0.651	0.698
	Demographic + Environmental factors	GLM	**0.709**	0.669 - 0.750	0.707	0.631	0.657	0.683
		RandomForest	**0.696**	0.657 - 0.735	0.702	0.626	0.652	0.677
	Demographic + GPS	GLM	**0.580**	0.539 - 0.622	0.573	0.506	0.537	0.542
		RandomForest	**0.576**	0.535 - 0.618	0.552	0.534	0.542	0.544
	Demographic only	GLM	**0.549**	0.517 - 0.582	0.521	0.571	0.548	0.544
		RandomForest	**0.552**	0.520 - 0.584	0.532	0.546	0.540	0.538
**Suicidal Ideation**	Demographic + GPS + Environmental factors	GLM	**0.750**	0.713 - 0.788	0.705	0.620	0.650	0.678
		RandomForest	**0.741**	0.704 - 0.778	0.731	0.603	0.648	0.692
	Demographic + Environmental factors	GLM	**0.732**	0.693 - 0.771	0.701	0.606	0.640	0.670
		RandomForest	**0.727**	0.690 - 0.764	0.695	0.623	0.648	0.671
	Demographic + GPS	GLM	**0.600**	0.559 - 0.641	0.573	0.529	0.549	0.553
		RandomForest	**0.575**	0.533 - 0.617	0.543	0.552	0.548	0.547
	Demographic only	GLM	**0.585**	0.551 - 0.619	0.492	0.567	0.532	0.527
		RandomForest	**0.591**	0.558 - 0.623	0.499	0.578	0.542	0.536
**Suicide Attempt**	Demographic + GPS + Environmental factors	GLM	**0.768**	0.640 - 0.895	0.582	0.591	0.587	0.586
		RandomForest	**0.717**	0.565 - 0.868	0.803	0.698	0.727	0.780
	Demographic + Environmental factors	GLM	**0.552**	0.396 - 0.707	0.658	0.641	0.647	0.652
		RandomForest	**0.714**	0.554 - 0.873	0.829	0.650	0.703	0.792

© 2022 Joo YY et al. *JAMA Network Open*.

	Demographic + GPS	GLM	**0.645**	0.488 - 0.801	0.530	0.517	0.523	0.524
		RandomForest	**0.629**	0.481 - 0.777	0.525	0.547	0.537	0.535
	Demographic only	GLM	**0.643**	0.536 - 0.749	0.587	0.475	0.528	0.535
		RandomForest	**0.646**	0.546 - 0.746	0.598	0.430	0.512	0.517

© 2022 Joo YY et al. *JAMA Network Open*.

eTable 7. Feature Importance of Elastic Net Model for Prediction of Suicidal Thoughts and Behaviors Among Youths

(A) European-ancestry individuals only

Overall Suicidality				Suicidal Ideation				Suicide Attempt			
**Demographic + GPS + Environmental factors**		**Demographic + GPS**		**Demographic + GPS + Environmental factors**		**Demographic + GPS**		**Demographic + GPS + Environmental factors**		**Demographic + GPS**	
	**Overall**		**Overall**		**Overall**		**Overall**		**Overall**		**Overall**
Depress.CBCL	100	married3	100	Depress.CBCL	100	sex1	100	TotProb.CBCL	100	income	100
Internal.CBCL	95.58	sex1	97.49	AnxDep.CBCL	98.76	married3	95.32	fes_q1	56.357	sex1	56.31
TotProb.CBCL	93.37	RISK4PC	76.06	Internal.CBCL	97.63	income	86.75	External.CBCL	48.8013	ADHD	45.39
External.CBCL	85.81	ADHD	65.3	TotProb.CBCL	89.11	GENERALHAPPINESS_MEANINGFUL	80.93	SCZ	35.9829	SNORING	31.71
AnxDep.CBCL	81.67	income	64.94	External.CBCL	82.56	SNORING	72.47	fes_q6	17.6458	IQ	28.09
Obsessive.Compulsive.Problems..OCD..CBCL	62.43	married5	50.65	Obsessive.Compulsive.Problems..OCD..CBCL	72.67	married5	68.77	PTSD	7.8757	race.ethnicity2	24.74
RuleBreak.CBCL	61.78	PTSD	43.84	fes_q4	65.69	SMOKER	56.92	married6	5.7067	married3	12.42
Conduct.CBCL	60.3	AD	39.82	Conduct.CBCL	58.78	CP	51.41	Sluggish.Cognitive.Tempo..SCT..CBCL	3.686	GENERALHAPPINESS	0
Stress.CBCL	59.24	GENERALHAPPINESS_MEANINGFUL	37.88	Stress.CBCL	58.62	RISKTOL	49.24	ASD	0.6916	race.ethnicity5	0
Opposit.CBCL	57.2	NEUROTICISM	37.69	Opposit.CBCL	56.35	PTSD	46.68	DEPRESSION	0	PTSD	0

© 2022 Joo YY et al. *JAMA Network Open*.

(B) Entire multiethnic individuals

**Overall Suicidality**				**Suicidal Ideation**				**Suicide Attempt**			
**Demographic + GPS + Environmental factors**		**Demographic + GPS**		**Demographic + GPS + Environmental factors**		**Demographic + GPS**		**Demographic + GPS + Environmental factors**		**Demographic + GPS**	
	**Overall**		**Overall**		**Overall**		**Overall**		**Overall**		**Overall**
Depress.CBCL	100	married3	100	Depress.CBCL	100	sex1	100	Internal.CBCL	100	income	100
AnxDep.CBCL	74.58	income	68.06	TotProb.CBCL	95.13	married3	99.24	AnxDep.CBCL	89.66	sex1	4.672
Internal.CBCL	71.78	married5	65.08	Internal.CBCL	91.86	race.ethnicity5	80.95	IQ	89.37	race.ethnicity3	0
sex1	66.44	sex1	56.29	AnxDep.CBCL	89.48	MDD	70.68	Obsessive.Compulsive.Problems..OCD..CBCL	87.56	married6	0
fes_q1	63.42	married6	53.23	External.CBCL	89.16	SMOKER	68.24	fes_q1	80.35	HAPPINESS	0
TotProb.CBCL	61.86	MDD	53	fes_q1	87.88	SNORING	66.66	TotProb.CBCL	77.08	NEUROTICISM	0
External.CBCL	61.18	age	51.8	fes_q3	72.17	GENERALHAPPINESS_MEANINGFUL	63.68	income	76.97	GENERALHAPPINESS_HEALTH	0
Opposit.CBCL	52.02	GENERALHAPPINESS_MEANINGFUL	49.48	fes_q2	71	age	55.5	Sluggish.Cognitive.Tempo..SCT..CBCL	72.77	DRINK	0
ADHD	50.2	INSOMNIA	47.4	Social.CBCL	64.64	income	51.32	fes_q2	72.05	age	0
Aggressive.CBCL	47.3	SMOKER	43.13	Obsessive.Compulsive.Problems..OCD..CBCL	62.57	ASD	46.59	External.CBCL	68.78	CP	0

© 2022 Joo YY et al. *JAMA Network Open*.

eTable 8. Early Life Stress Scale in Detail

Main-scale	Sub-scale		Title of scales	Contents of questions	Scoring	Meaning behind scoring		Make sub-scales	Make main-scales	Make ELS total
Abuse (Ab)	Family Environment Scale (parent and youth) KSADS-5, PTSD module	ABCD Youth Family Environment Scale-Family Conflict Subscale Modified from PhenX (FES)	ABCD Youth Family Environment Scale-Family Conflict Subscale Modified from PhenX (FES)	Family members sometimes hit each other.	0;1	0 = False; 1 = True	0; 1 (_PA_1, z-standardized)	Ab_PA = {(_PA_1) + (_PA_2)} / 2	Ab = (Ab_PA + Ab_SA)/2	ELS_total = (Ab + HC + Ne) / 3
		ABCD Parent Diagnostic Interview for DSM-5 (KSADS) Traumatic Events	ABCD Parent Diagnostic Interview for DSM-5 (KSADS) Traumatic Events	A family member threatened to kill your child	0;1	0 = No; 1 = Yes	0 - 12 (_PA_2, z-standardized)			
				A non-family member threatened to kill your child	0;1					
				Beaten to the point of having bruises by a grown up in the home	0;1					

© 2022 Joo YY et al. *JAMA Network Open*.

	Shot, stabbed, or beaten brutally by a grown up in the home	0;1
	Shot, stabbed, or beaten brutally by a non-family member	0;1
	Witnessed someone shot or stabbed in the community	0;1
	Witnessed death or mass destruction in a war zone	0;1
	Witnessed or present during an act of terrorism (e.g., Boston marathon bombing)	0;1

© 2022 Joo YY et al. *JAMA Network Open*.

			Witnessed or caught in a natural disaster that caused significant property damage or personal injury	0;1			
			Witnessed or caught in a fire that caused significant property damage or personal injury	0;1			
			Another significant accident for which your child needed specialized and intensive medical treatment	0;1			
			A car accident in which your child or another person in the car was hurt bad enough to require medical attention	0;1			
KSADS-5, PTSD module	ABCD Parent Diagnostic	ABCD Parent Diagnostic Interview for DSM-5	A peer forced your child to do something sexually	0;1	0 = No; 1 = Yes	0 - 3 (Ab_SA, z-standardized)	Ab_SA

© 2022 Joo YY et al. *JAMA Network Open*.

		Interview for DSM-5 (KSADS) Traumatic Events	(KSADS) Traumatic Events	An adult outside your family touched your child in his or her privates, had your child touch their privates or did other sexual things to your child	0;1					
				A grown up in the home touched your child in his or her privates, had your child touch their privates, or did other sexual things to your child	0;1					
Household Challenges	KSADS-5, PTSD module	ABCD Parent Diagnostic Interview for DSM-5 (KSADS) Traumatic Events	ABCD Parent Diagnostic Interview for DSM-5 (KSADS) Traumatic Events	Witness the grownups in the home push, shove or hit one another	0;1	0 = No; 1 = Yes	0; 1 (HC_MTV, z-standardized)	HC_MTV		HC = (HC_MTV + HC_HSA + HC_MIH + HC_PSD + HC_CHM) / 5

© 2022 Joo YY et al. *JAMA Network Open*.

Family History Assessment Adult Self-report (parent)	ABCD Family History Assessment Part 1	ABCD Family History Assessment Part 1	Has ANY blood relative of your child ever had any problems due to alcohol, such as: Marital separation or divorce; Laid off or fired from work; Arrests or DUIs; Alcohol harmed their health; In an alcohol treatment program; Suspended or expelled from school 2 or more times; Isolated self from family, caused arguments or were drunk a lot.	0;1	0 = No; 1 = Yes	0 - 336 [1] (_HSA_1, z-standardized)	HC_HSA = (_HSA_1+_HSA_2+_HSA_3+_HSA_4+_HSA_5+_HSA_6+_HSA_7) / 7

© 2022 Joo YY et al. *JAMA Network Open*.

			Has ANY blood relative of your child ever had any problems due to drugs, such as: Marital separation or divorce; Laid off or fired from work; Arrests or DUIs; Drugs harmed their health; In a drug treatment program; Suspended or expelled from school 2 or more times; Isolated self from family, caused arguments or were high a lot.	0;1	0 = No; 1 = Yes	0 - 336 [2] (_HSA_2, z-standardized)
	ABCD Parent Adult Self Report Scores Aseba (ASR)	ABCD Parent Adult Self Report Scores Aseba (ASR)	I use drugs (other than alcohol, nicotine) for nonmedical purposes	0;1;2	0 = Not True; 1 = Somewhat/Sometimes True; 2 = Very True/Often	0 - 2 (_HSA_3, z-standardized)
			I drink too much alcohol or get drunk	0;1;2		0 - 2 (_HSA_4, z-standardized)
			In the past 6 months, about how many times per day did you use tobacco (including smokeless tobacco)?	0 :: 100		0 - 2 (_HSA_5, z-standardized)

© 2022 Joo YY et al. *JAMA Network Open*.

				In the past 6 months, on how many days were you drunk?			0 - 180 (_HSA_6, z-standardized)	
				In the past 6 months, on how many days did you use drugs for nonmedical purposes (including marijuana, cocaine, and other drugs, except alcohol and nicotine)?	0 :: 180		0 - 180 (_HSA_7, z-standardized)	
	Family History Assessment (parent) Adult Self-report (parent)	ABCD Family History Assessment Part 1 ABCD Family History Assessment Part 2	ABCD Family History Assessment Part 1	Has ANY blood relative of your child ever suffered from depression, that is, have they felt so low for a period of at least two weeks that they hardly ate or slept or couldn't work or do whatever they usually do?	0;1	0 = No; 1 = Yes	0 - 48 [3] (_MIH_1, z-standardized	HC_MIH = (_MIH_1+_MIH_2+_MIH_3+_MIH_4+_MIH_5+_MIH_6+_MIH_7+_MIH_8+_MIH_9+_MIH_10+_MIH_11+_MIH_12) / 12
			ABCD Family History Assessment Part 2	Has ANY blood relative of your child ever had a period of time when others were concerned because they suddenly became more active day and night and seemed not to need any sleep and talked much more than usual for them?	0;1		0 - 48 [3] (_MIH_2, z-standardized	

© 2022 Joo YY et al. *JAMA Network Open*.

			Has ANY blood relative of your child ever had a period lasting six months when they saw visions or heard voices or thought people were spying on them or plotting against them?	0;1		0 - 48 [3] (_MIH_3, z-standardized)
			Has ANY blood relative of your child ever attempted or committed suicide?	0;1		0 - 48 [3] (_MIH_4, z-standardized)
	ABCD Parent Adult Self Report Raw Scores Aseba (ASR)	ABCD Parent Adult Self Report Raw Scores Aseba (ASR)	Depressive Problems ASR DSM-5-Oriented Scale (t score)	50 :: 100		0 - 28 (_MIH_5, z-standardized)
			Anxiety Problems ASR DSM-5-Oriented Scale (t score)	50 :: 80		0 - 12 (_MIH_6, z-standardized)
			Somatic Problems ASR DSM-5-Oriented Scale (t score)	50 :: 100		0 - 18 (_MIH_7, z-standardized)
			Avoidant Personality Problems ASR DSM-5-Oriented Scale t score)	50 :: 90		0 - 14 (_MIH_8, z-standardized)

© 2022 Joo YY et al. *JAMA Network Open*.

			AD/H Problems ASR DSM-5-Oriented Scale (t score)	50 :: 100		0 - 26 (_MIH_9, z-standardized)	
			Antisocial Personality Problems ASR DSM-5-Oriented Scale (t score)	50 :: 100		0 - 40 (_MIH_10, z-standardized)	
			Inattention ASR DSM-5-Oriented Scale (t score)	50 :: 90		0 - 14 (_MIH_11, z-standardized)	
			Hyperactivity-Impulsivity ASR DSM-5-Oriented Scale (t score)	50 :: 80		0 - 12 (_MIH_12, z-standardized)	
Demographics Survey (parent)	ABCD Parent Demographics Survey	ABCD Parent Demographics Survey	Are you now married, widowed, divorced, separated, never married or living with a partner?	1 :: 6	1= divorced, separated; 0 = the others	0; 1 (HC_PSD, z-standardized)	HC_PSD
Family history Assessment (parent)	ABCD Family History Assessment Part 2	ABCD Family History Assessment Part 2	Has ANY blood relative of your child been the kind of person who never holds a job for long, or gets into fights, or gets into trouble with the police from time to time, or had any trouble with the law as a child or an adult?	0;1	0 = No; 1 = Yes	0 - 48 [3] (HC_CHM, z-standardized)	HC_CHM

© 2022 Joo YY et al. *JAMA Network Open*.

Neglect	CRPBI Acceptance Subscale (youth)	ABCD Children's Report of Parental Behavioral Inventory	ABCD Children's Report of Parental Behavioral Inventory	First caregiver (caregiver participating in study/completing protocol). Makes me feel better after talking over my worries with him/her	1::3	1 = Not like him/her; 2 = Somewhat like him/her; 3 = A lot like him/her	(score reverse coding) 10 - 30(Ne_EN, z-standardized)	Ne_EN	Ne = (Ne_EN + Ne_PN) / 2
				First caregiver (caregiver participating in study/completing protocol). Smiles at me very often.	1::3				
				First caregiver (caregiver participating in study/completing protocol). Is able to make me feel better when I am upset.	1::3				
				First caregiver (caregiver participating in study/completing protocol). Believes in showing his/her love for me.	1::3				
				First caregiver (caregiver participating in study/completing protocol). Is easy to talk to.	1::3				

© 2022 Joo YY et al. *JAMA Network Open*.

			Second caregiver. Makes me feel better after talking over my worries with him/her.	1::3			
			Second caregiver. Smiles at me very often.	1::3			
			Second caregiver. Is able to make me feel better when I am upset.	1::3			
			Second caregiver. Believes in showing his/her love for me.	1::3			
			Second caregiver. Is easy to talk to.	1::3			
Parental Monitoring (youth)	ABCD Parental Monitoring Survey	ABCD Parental Monitoring Survey	How often do your parents/guardians know where you are?	1::5	1 = Never; 2 = Almost Never; 3 = Sometimes; 4 = Often; 5 = Always or Almost Always	(score reverse coding) 5 - 25 (Ne_PN, z-standardized)	Ne_PN
			How often do your parents know who you are with when you are not at school and away from home?	1::5			

© 2022 Joo YY et al. *JAMA Network Open*.

		If you are at home when your parents or guardians are not, how often do you know how to get in touch with them?	1::5
		How often do you talk to your mom/dad or guardian about your plans for the coming day, such as your plans about what will happen at school or what you are going to do with friends?	1::5
		In an average week, how many times do you and your parents/guardians, eat dinner together?	1::5

[1] 336 = max. 48 family members multiplied by max. 7 problems. Max. 48 people; paremtnal grandfather, paternal grandmother, biological mother, maternal grandfather, maternal grandmother, biological father, paternal uncle (max. 5 people), maternal uncle (max. 5 people), paternal aunt(max. 5 people), maternal aunt (max. 5 p eople), full sibling-younger (max. 5 people), full sibling-older (max. 5 people), half sibling-younger (max. 5 people), half sibling-older (max. 5 people), full sibling-same age (max. 2 people). Max. 7 problems; 1) Marital separation or divorce 2) Laid off or fired from work 3) Arrests or DUIs 4) Alcohol harmed their health 5) In an alcohol treatment program 6) Suspended or expelled from school 2 or more times 7) Isolated self from family, caused arguments or were drunk a lot.

[2] 336 = max. 48 family members multiplied by max. 7 problems. Max. 48 people; paremtnal grandfather, paternal grandmother, biological mother, maternal grandfather, maternal grandmother, biologica l father, paternal uncle (max. 5 people), maternal uncle (max. 5 people), paternal aunt(max. 5 people), maternal aunt (max. 5 p eople), full sibling-younger (max. 5 people), full sibling-older (max. 5 people), half sibling-younger (max. 5 people), half sibling-older (max. 5 people), full sibling-same age (max. 2 people). Max. 7 problems; 1) Marital separation or divorce 2) Laid off or fired from work 3) Arrests or DUIs 4) Drugs harmed their health 5) In a drug treatment program 6) Suspended or expelled from school 2 or more times 7) Isolated sel f from family, caused arguments or were high a lot

[3] 48 = max. 48 family members. Max. 48 people; paremtnal grandfather, paternal grandmother, biological mother, maternal grandfather, maternal grandmother, biologica l father, paternal uncle (max. 5 people), maternal uncle (max. 5 people), paternal aunt(max. 5 people), maternal aunt (max. 5 peopl e), full sibling-younger (max. 5 people), full sibling-older (max. 5 people), half sibling-younger (max. 5 people), half sibling-older (max. 5 people), full sibling-same age (max. 2 people).

© 2022 Joo YY et al. *JAMA Network Open*.

eFigure 1. Biplot From Principal Component Analysis of the Combined Genotype Data of 1000-Genome Reference Panel (Phase 3, Release 5) and ABCD Study Participants

Colored by self-reported ethnicity. We removed the outliers fell outside of the 6SD limits (blue line) from the center in PC spaces.

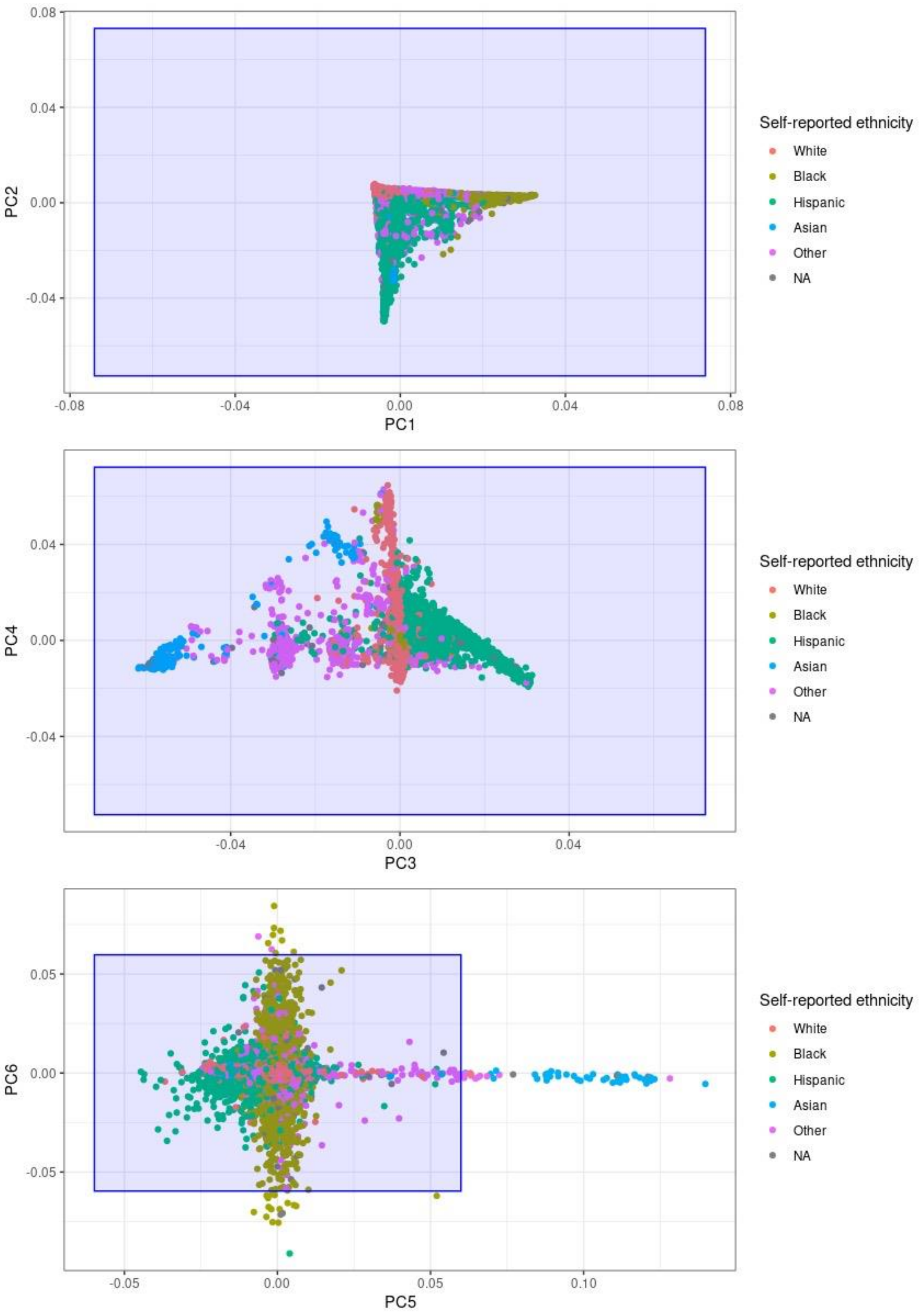

© 2022 Joo YY et al. *JAMA Network Open*.

eFigure 2. Analysis of the Association Between Multitrait Genome-Wide Polygenic Scores and Suicidal Thoughts and Behaviors Among 6592 Multiethnic Children

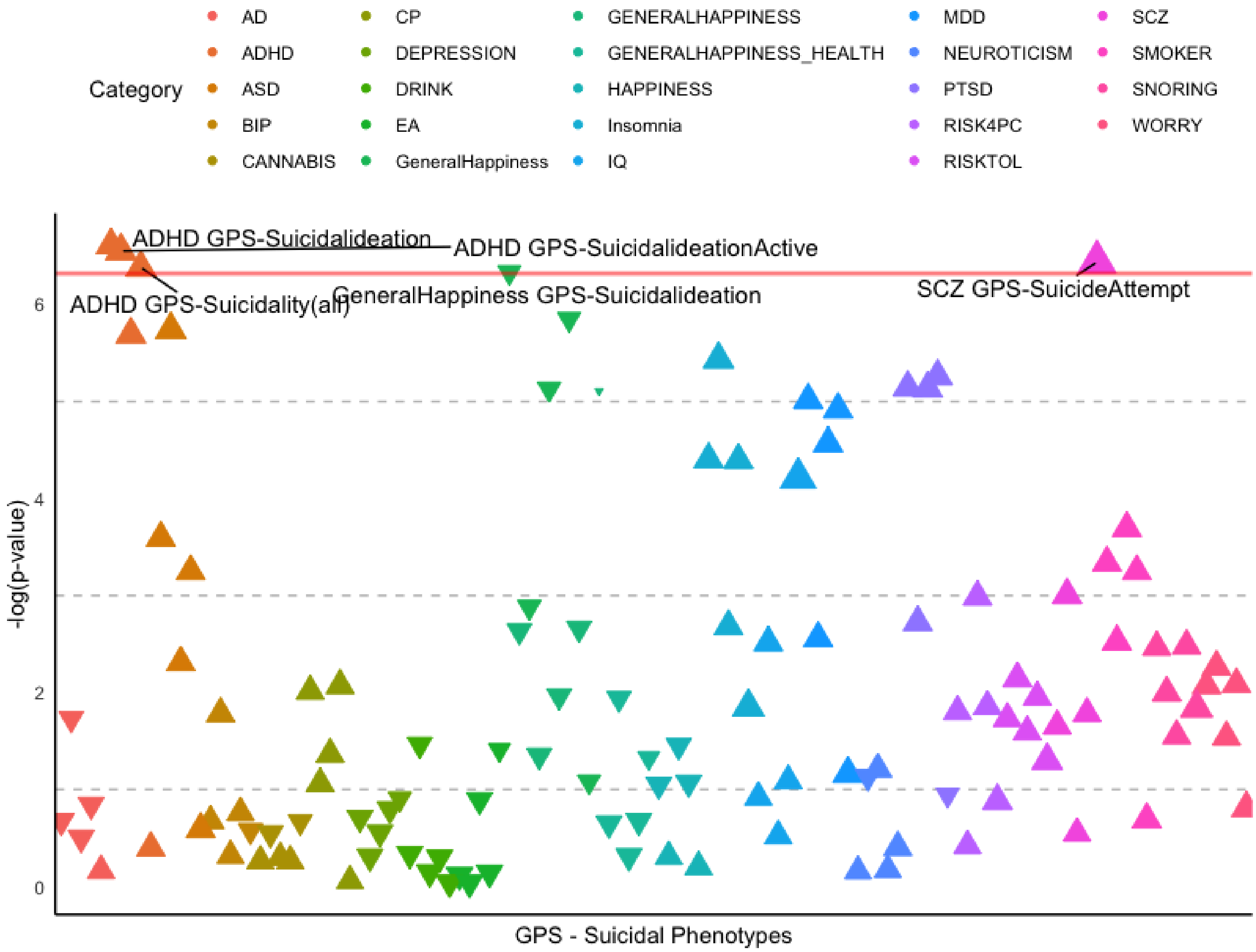

© 2022 Joo YY et al. *JAMA Network Open*.

eReferences

1. Conomos, M. P., Miller, M. B. & Thornton, T. A. Robust inference of population structure for ancestry prediction and correction of stratification in the presence of relatedness. *Genet. Epidemiol.* **39**, 276–293 (2015).

2. Conomos, M. P., Reiner, A. P., Weir, B. S. & Thornton, T. A. Model-free Estimation of Recent Genetic Relatedness. *Am. J. Hum. Genet.* **98**, 127–148 (2016).

© 2022 Joo YY et al. *JAMA Network Open*.
